# Combined Use of Chitosan and Olfactory Mucosa Mesenchymal Stem/Stromal Cells to Promote Peripheral Nerve Regeneration *In Vivo*

**DOI:** 10.1155/2021/6613029

**Published:** 2021-01-02

**Authors:** Rui D. Alvites, Mariana V. Branquinho, Ana C. Sousa, Irina Amorim, Rui Magalhães, Filipa João, Diogo Almeida, Sandra Amado, Justina Prada, Isabel Pires, Federica Zen, Stefania Raimondo, Ana L. Luís, Stefano Geuna, Artur S. P. Varejão, Ana C. Maurício

**Affiliations:** ^1^Departamento de Clínicas Veterinárias, Instituto de Ciências Biomédicas de Abel Salazar (ICBAS), Universidade do Porto (UP), Rua de Jorge Viterbo Ferreira, No. 228, 4050-313 Porto, Portugal; ^2^Centro de Estudos de Ciência Animal (CECA), Instituto de Ciências, Tecnologias e Agroambiente da Universidade do Porto (ICETA), Rua D. Manuel II, Apartado 55142, 4051-401 Porto, Portugal; ^3^Departamento de Patologia e Imunologia Molecular, Instituto de Ciências Biomédicas de Abel Salazar (ICBAS), Universidade do Porto (UP), Rua de Jorge Viterbo Ferreira, No. 228, 4050-313 Porto, Portugal; ^4^Instituto de Investigação e Inovação em Saúde (i3S), Universidade do Porto, R. Alfredo Allen, 200-135 Porto, Portugal; ^5^Institute of Molecular Pathology and Immunology of the University of Porto (IPATIMUP), 4200-465 Porto, Portugal; ^6^Universidade Católica Portuguesa, Centro de Biotecnologia e Química Fina (CBQF)-Laboratório Associado, Escola Superior de Biotecnologia, Rua Diogo Botelho 1327, 4169-005 Porto, Portugal; ^7^Centro Interdisciplinar de Estudo da Performance Humana (CIPER), Faculdade de Motricidade Humana (FMH), Universidade de Lisboa (ULisboa), 1495-751 Cruz Quebrada, Portugal; ^8^Instituto Politécnico de Leiria, Unidade de Investigação em Saúde da Escola Superior de Saúde de Leiria (UIS-IPL), Portugal; ^9^Centre for Rapid and Sustainable Product Development (CDrsp), Rua de Portugal, 2430-028 Marinha Grande, Portugal; ^10^Centro de Ciência Animal e Veterinária (CECAV), Universidade de Trás-os-Montes e Alto Douro (UTAD), Quinta de Prados, 5001-801 Vila Real, Portugal; ^11^Department of Clinical and Biological Sciences, Neuroscience Institute Cavalieri Ottolenghi, University of Turin, Regione Gonzole 10, 10043 Orbassano, Italy; ^12^Departamento de Ciências Veterinárias, Universidade de Trás-os-Montes e Alto Douro (UTAD), Quinta de Prados, 5001-801 Vila Real, Portugal

## Abstract

Peripheral nerve injury remains a clinical challenge with severe physiological and functional consequences. Despite the existence of multiple possible therapeutic approaches, until now, there is no consensus regarding the advantages of each option or the best methodology in promoting nerve regeneration. Regenerative medicine is a promise to overcome this medical limitation, and in this work, chitosan nerve guide conduits and olfactory mucosa mesenchymal stem/stromal cells were applied in different therapeutic combinations to promote regeneration in sciatic nerves after neurotmesis injury. Over 20 weeks, the intervened animals were subjected to a regular functional assessment (determination of motor performance, nociception, and sciatic indexes), and after this period, they were evaluated kinematically and the sciatic nerves and cranial tibial muscles were evaluated stereologically and histomorphometrically, respectively. The results obtained allowed confirming the beneficial effects of using these therapeutic approaches. The use of chitosan NGCs and cells resulted in better motor performance, better sciatic indexes, and lower gait dysfunction after 20 weeks. The use of only NGGs demonstrated better nociceptive recoveries. The stereological evaluation of the sciatic nerve revealed identical values in the different parameters for all therapeutic groups. In the muscle histomorphometric evaluation, the groups treated with NGCs and cells showed results close to those of the group that received traditional sutures, the one with the best final values. The therapeutic combinations studied show promising outcomes and should be the target of new future works to overcome some irregularities found in the results and establish the combination of nerve guidance conduits and olfactory mucosa mesenchymal stem/stromal cells as viable options in the treatment of peripheral nerves after injury.

## 1. Introduction

Peripheral nerve fibers are extremely fragile and sensitive structures, susceptible to being easily damaged by crushing, compression, or transection traumas [1]. Due to their high prevalence, peripheral nerve injuries (PNI) are one of the most important medical problems among traumatic hospital patients. The injury of a peripheral nerve essentially affects motor and sensory activity, interrupting the connection between the central nervous system and the target organs and muscles in the respective body region. In the long run, these injuries lead to behavioral changes, mobility difficulties, changes in consciousness and spatial perception, and loss of cutaneous and joint sensitivity and culminate in lifelong disabilities [2, 3]. These injuries are hard to standardize and treat essentially due to the wide variations that can occur regarding the type, location, severity, and complexity. Over the years, different medical and surgical approaches have been developed to treat PNI, but despite this, it has not yet been possible to establish the ideal and transversal treatment for all types of injuries [4]. It is known that the future of PNI treatment will involve the use of multifactorial treatments to promote sensory and motor recovery, and the maintenance of neuromuscular junctions to stimulate the reinnervation of target muscles and organs after periods of denervation is the primary objective.

To standardize PNI, nerve injuries have been classified at different levels depending on their severity, functional consequences, and prognosis. This classification scheme facilitates not only the determination of the pathophysiological phenomenon associated with the injury but also the establishment of the ideal treatment. The commonly applied PNI classification is based on that established by the combined efforts of Seddon and Sunderland [5, 6]. Seddon established 3 levels of PNI based on the degree of demyelination, the type of axonal injury, and the degree of alteration of the nerve connective tissue (*endoneurium*, *perineurium*, and *epineurium*): neuropraxia, axonotmesis, and neurotmesis [5]. Later, Sunderland expanded the PNI severity degrees to 5, with the first degree corresponding to neuropraxia, the fifth corresponding to neurotmesis, and the three intermediates deriving from axonotmesis, based on the involvement of the different connective tissue envelopments [6]. Despite the creation of this classification method, the difficulty in fitting all types of injuries into these categories later led Mackinnon to introduce an additional injury degree, basically a mixed injury, probably the most common in real clinical situations [7, 8].

After the occurrence of the PNI, two successive events of nerve degeneration and regeneration occur [4, 9], and this complex process involves the deletion of the inflammatory process, the production and release of neurotrophic factors, the stimulation of axonal regrowth, and the survival of neurons. Schwann cells also play an essential role during the regenerative phase of Wallerian degeneration, dividing and proliferating to form Büngner bands that not only produce neurotrophic and tissue adhesion factors but also guide axonal regrowth and promote regeneration of myelin sheaths [10]. However, all these regenerative phenomena are slow due to their complexity, which leads to excessively prolonged denervation periods in the target organs and muscles, which often culminate in functional losses and irreversible neurogenic muscle inflammation and atrophy [11]. Thus, without prompt interventions and effective surgical and therapeutic approaches, PNI remains an intricate clinical challenge.

In neurotmesis injuries, the most serious ones, whenever a good vascular bed is observed and an approximation of nerve endings without tension is possible, the application of end-to-end (EtE) sutures remains the treatment of choice [12]. When this technique cannot be applied, transplantation of autologous nerves to avoid rejection in the recipient is the gold standard technique, although also associated with several disadvantages: donor site morbidity and additional loss of function, difficulties in finding sources of nerves matching in size and characteristics, and performance of additional incisions [13]. The use of nerve guidance conduits (NGCs) has begun to be explored as a way to guide the regeneration of axons towards the distal nerve top, avoiding the occurrence of common misdirecting phenomena, severe inflammatory reactions, and fibrosis. Different types of material have already been tested for the production of NGCs, from nonabsorbable materials to biodegradable ones. It is currently accepted that these materials should constitute a completely degradable matrix, which positively influences the regenerative phenomena without their biodegradation process having negative local or systemic effects [14]. Despite this, no material has yet been established that has all the desirable characteristics and undoubtedly promotes effective nerve regeneration.

Chitosan is one of the materials that has been studied and is particularly advantageous because of its natural origin (chitin in the shell of arthropods and cell membranes of fungi, yeasts, and other microorganisms) [15], being completely bioabsorbed and not producing toxic metabolites that interfere with the regenerative process. In addition, chitosan promotes cell adhesion and maintenance of cell and tissue viability, has antimicrobial characteristics, and can be easily modified chemically and enzymatically to release cytokines, antibiotics, and components of the extracellular matrix (ECM) in a controlled manner [4]. Preclinical studies carried out with this material have shown that chitosan promotes axonal regeneration, improves functional recovery, and prevents the occurrence of extensive scarring and the development of neuromas [16]. The number of studies in which chitosan-based NGCs were used is small, and as such there is little data on the functional outcomes associated with their application. Some studies using a chitosan-polyglycolic acid combination demonstrated good motor and sensory recoveries when NGCs were used to bridge long-distance defects of the median nerve [17, 18]. In 2014, a commercial NGC was launched to the market, consisting purely of chitosan, and called Reaxon® Nerve Guide (Medovent GmbH (Mainz, Germany)). Developed in accordance with the international standard DIN EN ISO 13485 [19], Reaxon® was initially launched as 30 mm long NGCs with 5 internal diameter options to adapt to different therapeutic needs. Among the most important characteristics of these NGCs, their high flexibility and resistance to collapse, nonadherent surface, malleability, and transparency that facilitate the insertion of nerve tops and the application of sutures stand out. In addition, Reaxon® has a positively charged internal surface that can interact with negatively charged cells and molecules, allowing their combined action in promoting nerve regeneration. In this way, Reaxon® can be applied in tubulization techniques after lesions of neurotmesis, bridging long gaps through direct sutures between the nerve tops and the biomaterial, but also protecting the nerve tops connected through tension-free EtE sutures [20, 21].

Olfactory mucosa mesenchymal stem/stromal cells (OM-MSCs) are mesenchymal stem cells (MSCs) whose niche is in the *lamina propria* of the olfactory mucosa. With ectodermal origin, these cells express neural cell-related genes [22, 23] and have been adequately identified and isolated in different species [22–28]. Studies that focus on OM-MSCs are still few but have already shown that these cells have the minimum characteristics necessary for their characterization as MSCs [29]: plastic adherence in culture with the formation of fibroblastic-like colonies, capacity for tridifferentiation, and expression of specific surface markers with no expression of hematopoietic markers [22, 23]. Furthermore, these cells are also capable of following myogenic [30] and neurogenic differentiation [23, 30], and their conditioned medium is able to promote the proliferation of glial cells and activation of myelination *in vitro* [31]. Among the most important characteristics of OM-MSCs are their distribution in several areas of the nasal cavity that facilitates the collection in large and small animal models, both through *antemortem* and *postmortem* protocols, their origin in the neural crest, high versatility, chromosomal stability, and the ability to maintain self-renewal capacity over long periods of culture without changes associated with the donor's age [23, 32–34]. In *in vivo* studies, OM-MSCs were mostly applied in works associated with spinal cord trauma [35–37], degenerative diseases of the central nervous system [38, 39], lesions of the vestibulocochlear nerve [40–42], and lesions of the hippocampus [43, 44]. The studies in which these cells are applied to promote peripheral nerve regeneration after PNI are even more scarce, but the application of OM-MSCs in combination with a biphasic laminin and collagen-functionalized hyaluronic acid NGC has shown better outcomes than the use of the biomaterial alone [45].

ECM is the network of extracellular components whose function is to provide biochemical and structural support to cells, mainly consisting of macromolecules such as enzymes, glycoproteins, collagen, fibronectin, and laminin [46]. ECM is a fundamental component in the functional behavior of MSCs, since *in vivo* those are included in a three-dimensional microenvironment that interacts with neighboring tissues and responds to different types of signals, mediating cell attachment and migration [47, 48]. Hydrogels can be used to mimic the function of ECM in order to facilitate the formation and maturation of tissues in three-dimensional structures, both *in vitro* and *in vivo* [49]. Matrigel® is a gelatinous mixture of proteins, soluble and sterile, extracted from the basement membrane proteins derived from Engelbreth-Holm-Swarm mouse sarcomas and can be used as a basement membrane matrix for MSCs [50]. Its main component is laminin, also having collagen IV, proteoglycans, heparan sulfate, and entactin/nidogen in its constitution. Furthermore, it is also rich in several growth and proregenerative factors such as epidermal growth factor, insulin-like growth factor, fibroblast growth factor, TGF-*β*, and tissue plasminogen activator [51, 52]. At body temperature, 37°C, Matrigel® gels and forms a structure identical to three-dimensional ECM, supporting cell adhesion and differentiation and providing the ideal environment to promote *in vivo* regeneration of different tissues. Effectively, the use of Matrigel® promotes attachment and differentiation of neurons [53, 54] and supports *in vivo* peripheral nerve regeneration [55–58].

The combined use of MSCs with NGCs [59] and the combination of MSCs with Matrigel® [57] are two of the potential approaches to alter the paradigm associated with the stimulation of peripheral nerve regeneration after PNI. In this work, we tested the hypothesis that the combined use of Reaxon® NGCs and OM-MSCs suspended in Matrigel® may enhance the histomorphometric and functional regeneration of the rat's sciatic nerve after neurotmesis.

## 2. Materials and Methods

### 2.1. Preparation and Characterization of OM-MSCs

#### 2.1.1. Harvesting and Cell Culture

OM-MSCs were harvested from the rat's olfactory mucosa and maintained in culture as previously described [23, 60]. Briefly, OM-MSCs were collected from the rat's olfactory mucosa, nasal septum, and ethmoturbinates [60], isolated through a mixed method of enzymatic digestion and explant, expanded, and maintained in culture in standard conditions (37°C, 5% CO_2_ and humidified atmosphere) using a basal culture medium consisting of Basal Medium (DMEM/F12+GlutaMAX™ supplement, Gibco®) supplemented with 10% certified fetal bovine serum (One Shot Fetal Bovine Serum, Gibco®), 1.5% penicillin-streptomycin (Sigma-Aldrich®), and 1.5% amphotericin B (Sigma-Aldrich®). Cells were cryopreserved using basal culture medium and 10% dimethyl sulfoxide (DMSO) (Sigma-Aldrich®) in cryovials with at least 1 × 10^6^ cells. Before being used in the different assays, cells were thawed in a water bath (37°C), collected, centrifuged, resuspended in basal culture medium, counted, cultured, and maintained under standard conditions. For each assay, the culture medium was eliminated, the culture was washed with PBS, and the cells were detached using 0.25% Trypsin-EDTA (Sigma-Aldrich®) (3–5 min incubation under standard conditions). After centrifugation (1600 rpm, 10 min) and elimination of the supernatant, the number of cells and their viability were determined by a Trypan blue (Invitrogen™) exclusion cell assay using an automatic counter (Countess II FL Automated Cell Counter, Thermo Fisher Scientific®) [23].

#### 2.1.2. Reverse Transcriptase Polymerase Chain Reaction (RT-PCR)

To identify the expression of specific genes by OM-MSCs undifferentiated and after neurogenic differentiation, RT-PCR was performed.

*(1) RNA Isolation and cDNA Synthesis*. RNA isolation was carried out on OM-MSCs in their undifferentiated state and after the induction of neurogenic differentiation. Neurogenic differentiation was achieved as previously described [23]. For RNA isolation, the Aurum™ Total RNA Mini Kit (Bio-Rad Laboratories®) was used, following the manufacturer's instructions. Briefly, a 2 × 10^6^ cell pellet of both P6 differentiated (72 h of differentiation) and undifferentiated OM-MSCs was lysed with a lysis solution, followed by DNA removal with a DNAase I enzyme and elution with 80 *μ*l of an elution solution. RNA was maintained at -80°C.

Prior to cDNA synthesis, the purity and amount of the isolated RNA were determined using a UV-spectrophotometry technique and measuring the A_260_/A_280_ (to identify protein contamination) and A_260_/A_230_ (to identify phenol, polysaccharides, and/or chaotropic salt contaminations) absorbances on a NanoDrop™ One Microvolume UV-Vis Spectrophotometer (Thermo Scientific™). Purity values considered adequate include the interval 2-2.2 to A_260_/A_280_ and 1.8-2.2 to A_260_/A_230_ [61].

The first cDNA strands were synthesized from 4 *μ*l of total RNA in 20 *μ*l final volume, following the manufacturer's instructions for the iScript™ cDNA Synthesis Kit (Bio-Rad Laboratories®). The complete reaction mix was then incubated in the thermal cycler (T100™ Thermal Cycler, Bio-Rad Laboratories®) using time and temperature guidelines indicated by the manufacturer's instructions.

*(2) Quantitative RT-PCR Assay*. The RT-PCR assay was conducted using the CFX96 Touch™ Real-Time PCR Detection System (Bio-Rad Laboratories®) under standard PCR conditions and using iTaq™ Universal SYBR Green Supermix (Bio-Rad Laboratories®) according to the manufacturer's instructions. 22 genes associated with markers of neuroglial expression were searched, including specific markers of immature and mature neurons and markers of glial cells at different stages of maturation. Markers associated with glial cells include Aldh1l1, CD40, Cdh2, Cspg4, GAP43, GFAP, MPZ, NCAM, Nes, Ocln, Olig3, Sox10, and Sox2; neuron-associated markers include Ascl1, Dcx, MAP2, NueroD1, Rbfox3, Syn, Cdk5r1, Cript, Tubb3, and again GAP43. *β*-Actin was used as a housekeeping gene. A 96-well plate (PrimePCR Custom Plate 96 Well, Bio-Rad Laboratories®) was prepared with the 22 predesigned primers for the genes to be identified, in order to allow the identification of their genetic expression (Table S1 shows the list of genes under study with the respective amplicon context sequences, ensemble gene ID, and PCR product lengths). Plates were read in a real-time PCR detection system. Two pairs of primers targeting the genes were used to analyze their expression in undifferentiated and differentiated OM-MSCs. Plates containing the mix targeting the 22 genes were exposed to the temperature cycles indicated by the manufacturers. Once the RT-PCR was finished, the gene expression was analyzed and interpreted. To confirm the specificity of the product, the melting curves were also analyzed.

The values of threshold cycle (Ct) were interpreted as follows: Ct < 29 indicates strong positive reactions and abundant targeted nucleic acid in the sample, 30 < Ct < 39 indicates positive reactions and moderate amounts of targeted nucleic acid, and Ct > 39 appears in weak reactions with minimum targeted nucleic acid values or in environmental contaminations.

For the two groups of cells, the ΔCt value was calculated through the formula ΔCt = Ct_target gene_ − Ct_housekeeping gene_. Fold differences between the two groups were calculated using the standard ΔΔCt through the formula ΔΔCt = ΔΔCt_differentiated_ − ΔΔCt_undifferentiated_. The relative quantification (RQ) was determined by the formula RQ = 2^−(ΔΔCt)^. Genes with RQ values < 0.5 were considered downregulated, and those with RQ values > 2 were considered upregulated.

#### 2.1.3. Immunohistochemical Analysis

Early passage (P6) OM-MSCs were submitted to immunohistochemical analysis to detect specific antigens associated with neurogenic differentiation. In one plate, OM-MSCs were maintained in their undifferentiated state and in another; in the same passage, the cells were subjected to neurogenic differentiation as previously described [23]. In undifferentiated cells, culture was maintained until a confluence of 70–80% was reached; cells submitted to differentiation were maintained for 72 hours in the neurogenic differentiation medium. For both cases, the cells were detached using 0.25% Trypsin-EDTA solution and kept in a paraffin cytoblock (Surein® Preserve Cell solution®, Cytoglobe GmbH®). Subsequently, sections were cut at 2 *μ*m, subjected to deparaffinization, and dehydrated, followed by an immunohistochemical analysis using a Novolink™ Polymer Detection Systems (Leica Biosystems®) kit, according to the manufacturer's instructions. Information about the primary antibodies and antigen retrieval methods used is depicted in Table 1. The antibodies were selected to identify the presence of markers of neuronal (NeuN and GAP43) and glial (GFAP) origin, complementary to the results obtained by the RT-PCR technique.

Cellular immunoexpression was evaluated using an Eclipse E600 microscope (Nikon®) and the software Imaging Software NIS-Elements F Ver4.30.01 (Laboratory Imaging®), with respective interpretation and photographic record. For the different markers, immunoexpression was scored for labeling intensity (0, negative; +, weak; ++, moderate; and +++, strong). When distinct cytoplasm and nuclear staining was recognized in at least 5% of the total cell populations, immunoreactivity was considered positive.

### 2.2. *In Vitro* Cytocompatibility Assessment

To determine the compatibility between OM-MSCs and Reaxon® NGCs and Matrigel® to be used in the *in vivo* assays, a PrestoBlue™ viability assay was carried out. Four groups were established: (1) OM-MSCs seeded in contact with Reaxon® tubes and with basal medium, (2) OM-MSCs seeded on Matrigel® (Corning®) and with basal medium, (3) OM-MSCs seeded with basal medium (positive control), and (4) OM-MSCs seeded with basal medium supplemented with 10% DMSO (negative control) instead of 10% FBS. In the first group, Reaxon® NGCs were cut transversely to a suitable length and then placed inside a row of wells on a 24-well plate. In the second group, Matrigel® diluted in DMEM/F12 (1 : 1) was applied to a row of wells on the 24-well plate to cover its bottom, following the manufacturer's instructions. In the wells of all groups, OM-MSCs were seeded at a density of 6000 cells/cm^2^, with the addition of the medium considered. Cell metabolic activity was assessed at 24 h, 72 h, 120 h, 168 h, and 216 h, always in quadruplicate for each group in each time point. At each time point, the culture media were replaced with fresh complete medium, with the addition of 10% (*v*/*v*) of 10x PrestoBlue™ cell viability reagent (Invitrogen, A13262). Unseeded control wells were used as blank sets. Then, the plates were incubated for 1 hour under standard conditions. After this period, to identify supernatant color reflecting cell viability variances, the supernatant from each well was collected and transferred to a 96-well plate, followed by the absorbance reading at 570 nm (excitation wavelength) and 595 nm (emission wavelength) by spectrophotometry using a microplate photometer (Thermo Scientific™ Multiskan™ FC). Absorbance reading from each well was normalized to the emission wavelength, and the corrected absorbance value was obtained by the subtraction of the average of unseeded control wells to each experimental well. After collecting the supernatant to be analyzed, the wells were washed with PBS to remove the remains and residues of PrestoBlue™ dye and a new fresh medium was added. Between evaluations, the plates were kept in standard conditions.

### 2.3. Scanning Electron Microscopy Analysis

After determining the cytocompatibility between OM-MSCs and Reaxon®, a scanning electron microscopy (SEM) Analysis and energy-dispersive X-ray spectroscopy (EDS) exams were performed, using a high-resolution (Schottky) environmental scanning electron microscope with X-ray microanalysis and electron backscattered diffraction analysis: Quanta 400 FEG ESEM/EDAX Genesis X4M, operating in a high vacuum mode at an accelerating voltage of 15 kV SEM.

Reaxon® NGCs were cut transversely to fit the diameter of a well in a 24-well plate and then cut longitudinally. Each half-NGC was then placed inside a well. OM-MSCs were seeded in a density of 6000/cm^2^ with basal culture medium over the internal surface of the cut NGCs, remaining in culture for 216 hours, with medium changes every 2-3 days. After this period, the wells were washed three times with 0.1 M HEPES buffer (Merck®, PHG0001). The cells on the inner surface of the NGCs were fixed with 2% buffered glutaraldehyde (Merck®, G7651) and kept in this solution overnight. Then, the cells were washed in three cycles of five minutes with 0.1 M HEPES buffer, with gentle agitation. The samples were then dehydrated in a graded series of ethanol (50%-70%-90%-99%), followed by chemical drying with hexamethyldisilazane (HMDS) (Merck®, 440191) in ethanol for 15 minutes and an incubation with HMDS alone for 15 minutes. Finally, after removing HDMS from all wells, the plate was left overnight in the laminar flow chamber for total evaporation. Before SEM and EDS, samples collected from the wells were coated with gold/palladium by sputtering, using the SPI Module Sputter Coater equipment.

### 2.4. *In Vivo* Assays

#### 2.4.1. Animals

All steps involving animals were approved by the Organism Responsible for Animal Welfare (ORBEA) of the Abel Salazar Institute for Biomedical Sciences (ICBAS) from the University of Porto (UP) (project 209/2017) and by the Veterinary Authorities of Portugal (DGAV) (project DGAV: 2018-07-11014510), always in conformity with the Directive 2010/63/EU of the European Parliament and the Portuguese DL 113/2013, following the OECD Guidance Document on the Recognition, Assessment and Use of Clinical Signs as Humane Endpoints for Experimental Animals Used in Safety Evaluation (2000). Additionally, all measures were taken to avoid any pain and discomfort and to guarantee animal welfare, considering all humane endpoints for animal suffering and distress.

Thirty rats (*Rattus norvegicus*), Sasco Sprague-Dawley Breed, 8 to 9 weeks old, and 250-300 g BW (Charles River, Barcelona, Spain), were used in this work. Only males were used, to avoid hormonal influences resulting from the different reproductive phases observed in females. Animals were housed in rooms with controlled temperature and humidity, 2 to 3 animals per cage, with 12-12 h light/dark cycles. The cages were equipped with environmental enrichment, and the normal physiological activities were allowed, under standard laboratorial conditions. Chow and water were always available *ad libitum*. After reception, animals were kept under acclimatization for two weeks.

#### 2.4.2. Experimental Design

**Figure 1:**
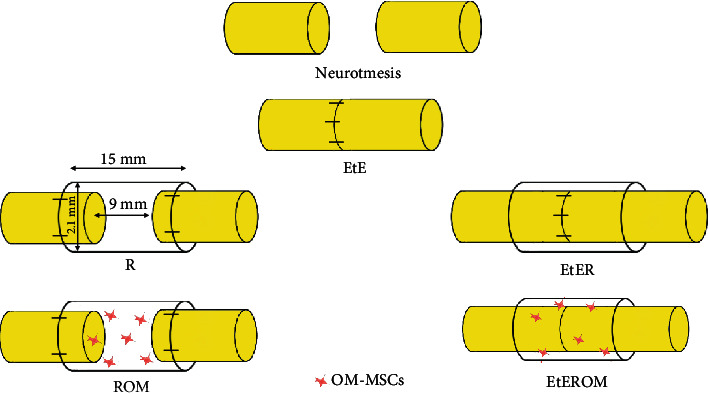
Experimental therapies applied to the sciatic nerve after neurotmesis injury. The applied NGCs are 15 mm long and have an internal diameter of 2.1 mm.

Rats submitted to a neurotmesis lesion of the sciatic nerve were divided into six experimental therapeutic groups (Figure 1): group 1—uninjured control (UC) (*n* = 30), group 2—EtE (*n* = 5), group 3—suture of the nerve ends to the Reaxon® NGC (R) (*n* = 6), group 4—EtE and wrapping with Reaxon® NGC (EtER) (*n* = 6), group 5—suture of the nerve ends to the Reaxon® NGC and administration of OM-MSCs suspended in Matrigel® (1 × 10^6^ cells suspended in 300 *μ*l of Matrigel® diluted in basal culture medium (1 : 1)) (ROM) (*n* = 7), and group 6—EtE, wrapping with Reaxon® NGC, and administration of OM-MSCs suspended in Matrigel® (EtEROM) (1 × 10^6^ cells suspended in 300 *μ*l of Matrigel® diluted in basal culture medium (1 : 1)) (*n* = 6). Reaxon® NGCs with 2.1 mm internal diameter and 15 mm length were used. Nerve transection and repair were performed on the right hind limb, and the contralateral limb was used as the uninjured control. Primary outcomes consisted of assessing motor, nociceptive, and behavioral function and kinematic evaluation; secondary outcomes included the determination of the mass of the cranial tibial muscle (*M. tibialis cranialis*) and its histomorphometric evaluation and also the stereological assessment of the sciatic nerve after collection.

#### 2.4.3. Surgical Procedures

The surgical techniques used in this work were based on the protocols used and described previously [62–64], and the specific procedures are below:

*(1) Sciatic Nerve Repair Model*. Animals were anesthetized with xylazine/ketamine (Rompun®/Imalgene 1000®; 1.25 mg/9 mg per 100 g BW), administered intraperitoneally. Once anesthetized, the rats were placed in left lateral decubitus and the right limb was prepared for surgery (trichotomy and asepsis). Surgeries were conducted under an M-650 operating microscope (Leica Microsystems, Wetzlar, Germany). The nerve was accessed through a skin incision initiated at the level of the greater trochanter and extending distally to midhalf of the thigh. After careful subcutaneous debridement, a separation of the *vastus lateralis* and *biceps femoris* muscles was performed to expose the sciatic nerve. With the muscles kept apart by a soft tissue retractor, the sciatic nerve was carefully separated from neighboring tissues and immobilized, followed by a complete transection (neurotmesis) with a straight microsurgical scissor immediately before the branching site into common peroneal and tibial nerves.

With the contralateral nerve being considered the control and being part of the UC group, the intervened nerves were subjected to the therapeutic approaches considered. In the EtE group, the nervous tops were coaptized, ensuring anatomical alignment and orientation and maintaining a minimum gap between the nerve ends. To ensure this position and to avoid misalignments and rotations, simple interrupted epineural microsutures between the two nervous tops (2-4 sutures) using 7/0 monofilament nylon were applied. In group R, about 3 mm of the nerve tops was inserted into the Reaxon® NGCs and fixed to the biomaterial as aligned and oriented as possible through epineural microsutures, using 7/0 monofilament nylon (2-4 sutures) and leaving a gap of approximately 10 mm between the two nerve ends. In the EtER group, tension-free suture was applied as described. Then, Reaxon® NGCs, with a longitudinal incision, were placed wrapping the nerve and the suture site, with the nerve and NGC being properly accommodated among the muscle mass to avoid displacement of the biomaterial. In the ROM group, the nerve tops were inserted into Reaxon® NGC and sutured as described. Then, OM-MSCs suspended in culture medium and Matrigel® were injected inside the NGC to fill its internal diameter. Finally, in the EtEROM group, the application of tension-free suture and wrapping with Reaxon® NGC was carried out as described, followed by the filling of the internal diameter of the NGC with OM-MSCs suspended in culture medium and Matrigel®. After the surgical intervention, the muscular, subcutaneous, and cutaneous layers were closed with simple interrupted sutures with 4-0 absorbable material. To avoid autotomy, all animals received a deterrent substance on the right paw. Anesthetic recovery was monitored in all animals, and the cages were returned to the housing facilities after all animals had awakened and returned to their normal behavior. After surgery, and for five consecutive days, the animals were medicated with carprofen (2-5 mg/kg SC QD). Throughout the study period, animals were frequently monitored and evaluated for signs of autotomy, contractures, and the development of wounds or infections.

#### 2.4.4. Functional Assessment

After the surgical performance of standardized neurotmesis injuries and application of therapeutic approaches, a periodic functional evaluation was carried out to determine the progression of the nervous regenerative process. All animals were evaluated before surgery (week 0) to establish the baseline of healthy behavior. After neurotmesis, the animals were evaluated after one and two weeks and thereafter every two weeks until week 20. All tests of the functional assessment sessions were performed by the same operator with experience in the applied techniques to avoid interindividual variances, and efforts were made to ensure a calm and stress-free environment so as not to condition the observed results.

**Figure 2:**
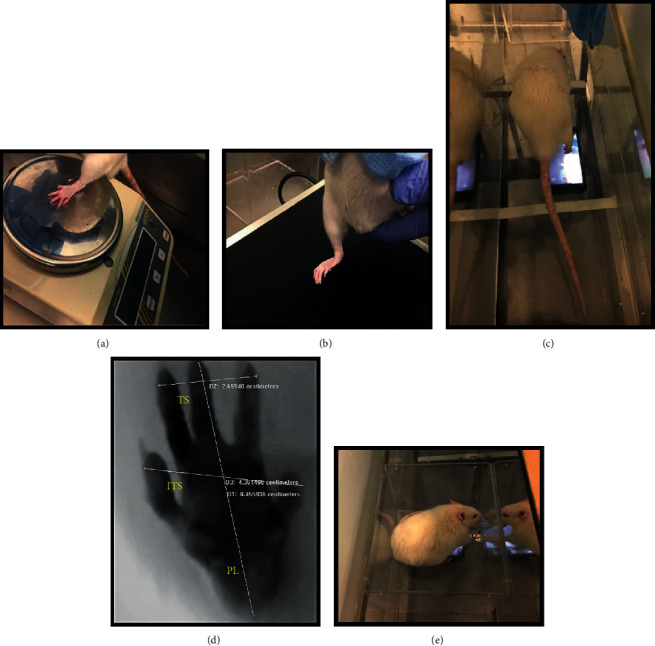
(a) Execution of the EPT test, with extension of the paw on the weighing machine to test motor performance. (b) Execution of the WRL test, with positioning of the right paw on the heating plate to test nociception. (c) Determination of the SFI with video recording of the animal's paw in contact with the acrylic corridor during gait. (d) Analysis of the animal's paw in contact with the acrylic corridor during gait, with determination of the PL, TS, and ITS values. (e) Determination of SSI with photographic record of the animal's paw in contact with the acrylic box. EPT: extended postural thrust; WRL: withdrawal reflex latency; SFI: sciatic functional index; SSI: static sciatic index; PL: print length; TS: toe spread; ITS: intermediate toe spread.

*(1) Motor Performance*. The extended postural thrust (EPT) test was used to assess motor performance, as previously described [62, 65]. To perform this test, the rat's body is wrapped in a soft cloth, keeping the head and hind limbs exposed. Then, the animal is suspended over a digital weighing machine (model TM 560; Gibertini, Milan, Italy) and lowered slowly towards it. It is important that the rat is able to maintain visual contact with the weighing machine in order to be able to anticipate contact and voluntarily extend the hind limb. Contact with the weighing machine must be made with the distal metatarsals and digits (Figure 2(a)). The force applied during contact with the weighing machine is measured in grams and recorded for both the healthy (NEPT) and injured (EEPT) limbs. The registration is done in triplicate, and the final value considered is the average of the three weighing values. The EEPT and NEPT values are then integrated into the following equation [66], in order to determine the percentage of motor functional deficit:

(1) $$\begin{array}{c} \%\text{motor deficit}=\frac{\text{NEPT}-\text{EEPT}}{\text{NEPT}}\times 100. \end{array}$$

*(2) Nociceptive Function*. The withdrawal reflex latency (WRL) test was used to assess the integrity of nociceptive function [67]. As in the previous test, the animal is wrapped in a soft cloth and suspended over a heating plate at 56°C (model 35-D, IITC Life Science Instruments, Woodland Hill, CA). The rat's paw should be placed over the surface of the heating plate, ensuring that the lateral aspect of the paw innervated by the sciatic nerve intervened is the one that contacts the plate [65]. WRL is defined as the time, in seconds, that it takes for the animal to retract its hind limb after contact of the corresponding paw with the surface of the plate. This time is determined by averaging three measurements for each limb, waiting two minutes between each measurement to avoid sensitization phenomena. In the case of a healthy nerve, the animal will retract the limb in 4.3 s or less. 12 s is considered the maximum limit of contact between the paw and the heating plate, and if the animal does not retract the limb in this period, the operator should move it away to avoid injuries and burns to the tissues [68]. The animal's paw should be placed gently on the plate, since manual compression can activate mechanoreceptors and cause the limb to retract without nociceptive activation [69] (Figure 2(b)). Similarly, it is important to remember that the medial aspect of the plantar surface of the paw is innervated by the saphenous nerve, branch of the femoral nerve, and as such saphenous nerve collateral sprouting around the denervated zone is possible. Therefore, it is important to ensure contact of the paw's lateral aspect against the heated surface to guarantee truthful results [65].

*(3) Walking Track Analysis*. The assessment of the walking pattern can be done by determining the sciatic functional index (SFI). In this work, a video collection technique was applied, adapting the techniques used in other groups using ink footprint records [65]. The animal is placed inside a transparent acrylic corridor, below which an image capture system is placed. At the end of the corridor, a shelter is installed to encourage the animal to walk in that direction. The rat is placed at the beginning of the tunnel and stimulated to move towards the shelter, allowing the registration of the feet in contact with the floor of the corridor when passing over the recording system (Figure 2(c)). For each animal, three records corresponding to three valid steps were considered, and a valid step is one in which paleness in the paw limits and support points are observed, resulting from its pressure against the floor of the corridor. The acquired images were then analyzed using an image processing software (Image-Pro Plus® 6, Media Cybernetics, Inc.), considering the calibration corresponding to the distance between the floor of the corridor and the video capture device and allowing the obtainment of the three measures considered for both healthy and experimental paws: print length (PL), toe spread distance between toes 1 and 5 (TS), and toe spread between toes 2 and 4 (ITS) (Figure 2(d)). The mean values obtained are then used in the following formulas (N stands for “normal” and E stands for “experimental”):

(2) $$\begin{array}{c} \text{Print lengh factor }\left(\text{PLF}\right)=\frac{\text{EPL}-\text{NPL}}{\text{NPL}},\\ \text{Toe spread factor }\left(\text{TSF}\right)=\frac{\text{ETS}-\text{NTS}}{\text{NTS}},\\ \text{Intermediate toe spread factor }\left(\text{ITF}\right)=\frac{\text{EIT}-\text{NIT}}{\text{NIT}}. \end{array}$$

The values collected are then introduced in the following formula:

(3) $$\begin{array}{c} \text{SFI}=\left(-38.3*\text{PLF}\right)+\left(109.5*\text{TSF}\right)+\left(13.3*\text{ITF}\right)-8.8. \end{array}$$

SFI values are around 0 on healthy animals and close to -100 on animals with a complete lesion in the sciatic nerve [70].

The static sciatic index (SSI) is derived from SFI but has the particularity of being determined in standing animals and does not consider the PL value [71]. For this measurement, the rat is placed inside a transparent acrylic box, which in turn is placed inside the corridor. Thus, the animal is stationary, with both limbs in contact with the box floor, allowing the photographic capture through the device placed immediately below (Figure 2(e)). The TS and ITS values of both healthy and experimental limbs are carefully measured in triplicate as previously described, and the mean values are integrated into the following formulas:

(4) $$\begin{array}{c} \text{Toe spread factor }\left(\text{TSF}\right)=\frac{\text{ETS}-\text{NTS}}{\text{NTS}},\\ \text{Intermediate toe spread factor }\left(\text{ITF}\right)=\frac{\text{EIT}-\text{NIT}}{\text{NIT}}. \end{array}$$

The SSI value is determined by the formula:

(5) $$\begin{array}{c} \text{SSI}=\left(108.44*\text{TSF}\right)+\left(31.85*\text{ITF}\right)-5.49. \end{array}$$

The SSI value is around -5 on healthy animals and around -90 on animals with complete sciatic nerve lesions [70].

**Figure 3:**
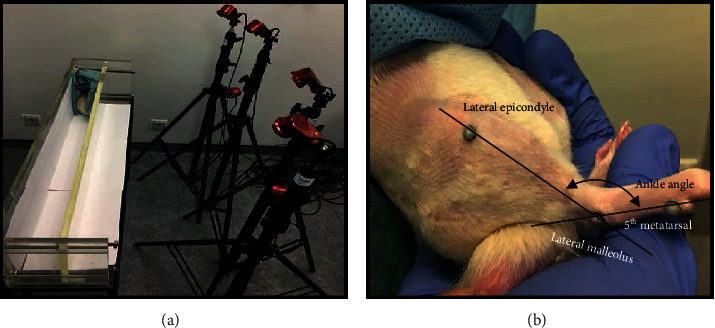
(a) Image capture set for kinematic analysis. At the end of the acrylic track, a shelter is placed to attract the animal, guaranteeing a straight line during locomotion. (b) Anatomical positions for placement of the reflective markers on the hind limb of the rat, with the respective ankle angle.

*(4) Kinematic Analysis*. Ankle kinematics during the gait cycle were recorded at the end of the 20 weeks for the established therapeutic groups and compared with the gait cycle pattern of the UC group, whose values were obtained before the induction of neurotmesis lesions. Motion capture was collected with an optoelectronic system of 6 cameras (OptiTrack Flex 3 model, NaturalPoint, Inc.) and the software Motive (NaturalPoint, Inc.) operating at a 120 Hz framerate. Animals walked on an acrylic track with length, width, and height of, respectively, 120, 12, and 15 cm (Figure 3(a)). After trichotomy, three reflective markers with 2 mm diameter were attached to 3 bony prominences on the right hind limb of the rat: the fifth metatarsal, the lateral malleolus, and the lateral epicondyle. All markers were placed by the same operator to avoid interindividual variations. Two body segments (foot and shank) were reconstructed using the Visual 3D software, and the rat's ankle angle (*θ*°) was determined using the scalar product between the vector representing the foot and the vector representing the shank (Figure 3(b)) during the stance phase and swing phase considered in the rat gait cycle [72]. With this model, positive and negative values for the position of the ankle joint indicate dorsiflexion and plantarflexion, respectively. All the gait variables were normalized for 100% of the gait cycle, and for each cycle, the initial contact and the toe-off instants were identified. The normalized temporal parameters were averaged over all recorded trials.

#### 2.4.5. Stereological and Histomorphometric Analysis

**Figure 4:**
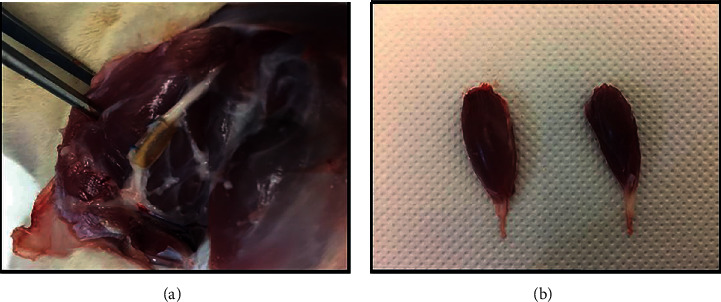
(a) Exposure of the sciatic nerve of the ROM therapeutic group, 20 weeks after surgery. It is possible to observe the regenerated nerve filling all the lumen of the NGC, with the two nerve tops connected. (b) Comparison of the mass of cranial tibial muscles of the healthy limb (left) and subject to neurotmesis (right) after collection at 20 weeks.

*(1) Nerve Stereological Analysis*. After the established 20 weeks of the study, animals were euthanized using general anesthesia followed by lethal overdose with Eutasil® (200 mg/ml, 200 mg/kg b.w., intraperitoneally). Once euthanasia was confirmed, the injured sciatic nerves (10 mm long samples distal to the lesion site) and uninjured controls were collected (Figure 4(a)) and adequately fixed for further stereological evaluation through light and electron microscopy. The access to the nerve to be collected was performed in the same way as access to surgical intervention. After exposure of the sciatic nerve, it was covered with fixation solution (2.5% purified glutaraldehyde and 0.5% saccharose in 0.1 M Sorensen phosphate buffer (pH 7.4, 4°C)) in order to promote its hardening and facilitate handling and collection. After excision, the 10 mm long segment whose proximal portion was properly identified was immersed in the same fixation solution and correctly aligned and oriented for 5 minutes to prevent nerve tangling. Then, the segments were kept immersed in the fixation solution for 6-8 hours, after which they were washed thoroughly and immersed in 1.5% saccharose wash solution in 0.1 M Sorensen phosphate buffer (pH 7.4) for 6-12 h. The histological preparation and histomorphometric evaluation were later performed according to the previously described protocol [73], and the parameters total number of fibers (*N*), fiber density (N/mm^2^), axon diameter (*μ*), fiber diameter (*μ*), myelin thickness (*M*, *μ*), and cross-sectional area (mm^2^) were considered, in addition to the ratios *d*/*D* (g-ratio). A systematic random sampling and D-dissector were adopted [74–77].

*(2) Histomorphometric Muscle Analysis*. In parallel with the collection of the sciatic nerves, the cranial tibial muscles were also collected (Figure 4(b)) for further histomorphometric assessment and determination of the neurogenic atrophy degree. The collected muscles from both injured and healthy limbs were weighed and then fixed with 4% buffered formaldehyde and processed for routine histopathological analysis (haematoxylin and eosin (H&E)). From the midbelly region of the muscle, consecutive sections (3 *μ*m thick) were obtained, prepared, and stained, and low-magnification images (100x) were obtained with a Nikon® microscope connected to a Nikon® digital camera DXM1200. The images obtained were processed using ImageJ© software (NIH), through an unbiased sampling procedure. Individual fiber measurements were obtained, and the muscle fiber area and minimal Feret's diameter (minimum distance of parallel tangents at opposing borders of the muscle fiber) were calculated. Measurements were blindly and randomly obtained from a minimum of 800 fibers for each group, by 2 independent and experienced operators.

#### 2.4.6. Statistical Analysis

Statistical analysis was performed using the software GraphPad Prism version 6.00 for Windows (GraphPad Software, La Jolla, California, USA). When appropriate, data were expressed as mean ± SEM. Comparisons between groups were performed with an analysis through a parametric test. A value of *p* < 0.05 is considered statistically significant. Significance of the results is shown according to *p* values by the symbol ∗: ∗ corresponds to 0.01 ≤ *p* < 0.05, ∗∗ to 0.001 ≤ *p* < 0.01, ∗∗∗ to 0.0001 ≤ *p* < 0.001, and ∗∗∗∗ to *p* < 0.0001.

For the kinematic analysis, in order to objectively quantify the differences in the gait analysis, a statistical parametric mapping (SPM) was used, a statistical approach that allows hypothesis testing on kinematic and kinetic waveforms without the need of a priori data reduction. SPM was used to analyze the ankle planar angle in gait trials, in a large cohort of rat after neurotmesis. SPM unpaired *t*-tests were performed, comparing the mean kinematic angle of each pattern to the respective mean kinematic angle of the control group (*α* = 0.05). All analyses were performed on retrospectively collected data, using open-source SPM1d version M.0.4.7 (2019.11.27; http://www.spm1D.org) in MATLAB1.

## 3. Results

### 3.1. RT-PCR

Ct average values for each gene, ΔCt, ΔΔCt, and RQ values can be seen in Table 2. The purity level assay using the UV-spectrophotometry technique revealed that all RNA samples were pure, allowing their use in the following stages. The analysis of the melting curves revealed the presence of a single peak in the curve of each gene, thus confirming the amplification of a single amplicon. In undifferentiated cells, the Cript and Cspg4 genes showed Ct < 29. All other genes, excluding Olig3, have Ct values between 29 and 39, indicating positive reaction and the presence of the target nucleic acid in the sample in moderate amounts. The Dcx, Rbfox3, and Syp genes were not identified in these cells, as well as the MPZ gene that was not identified in any of the cell groups. In differentiated cells, only Cript showed Ct < 29, with Ascl1, Dcx, and GAP43 with values > 39 and all other genes identified with positive reaction and in moderate amounts in the samples. The Ocln and Olig3 genes, identified in the undifferentiated cells, were not found in the differentiated ones. Conversely, the Dcx, Rbfox3, and Syp genes were identified in the cells only after neurogenic induction.

**Figure 5:**
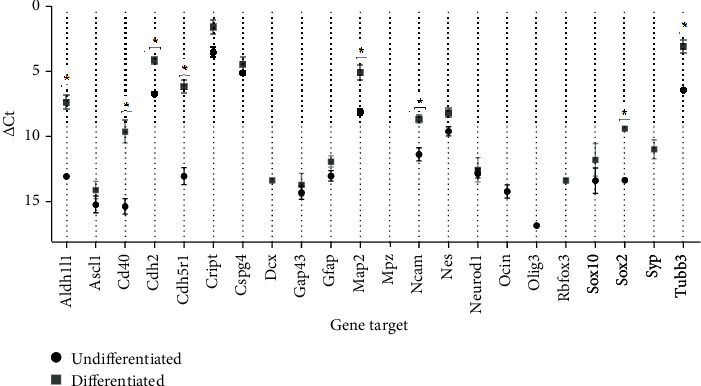
ΔCt values for the different genes under study in OM-MSCs undifferentiated and after neurogenic differentiation. Higher delta-CT values represent lower expression. Results are presented as mean ± SEM.

Comparing the two passages, changes in fold expression of the genes were observed. In the genes identified in the two types of cells, upregulation was observed in practically all of them in neurologically differentiated cells compared to undifferentiated ones, excluding Cspg4, GAP43, and Neurod1 that maintained normal expression. No downregulated genes were observed, and in genes that were not identified in the two cell types, it was not possible to determine the relative expression. Statistically significant differences were identified between ΔCt values of the genes Aldh1l1, Cd40, Cdh2, Cdk5r1, Map2, Ncam, Sox2, and Tubb3 (Figure 5).

### 3.2. Immunohistochemical Analysis

**Figure 6:**
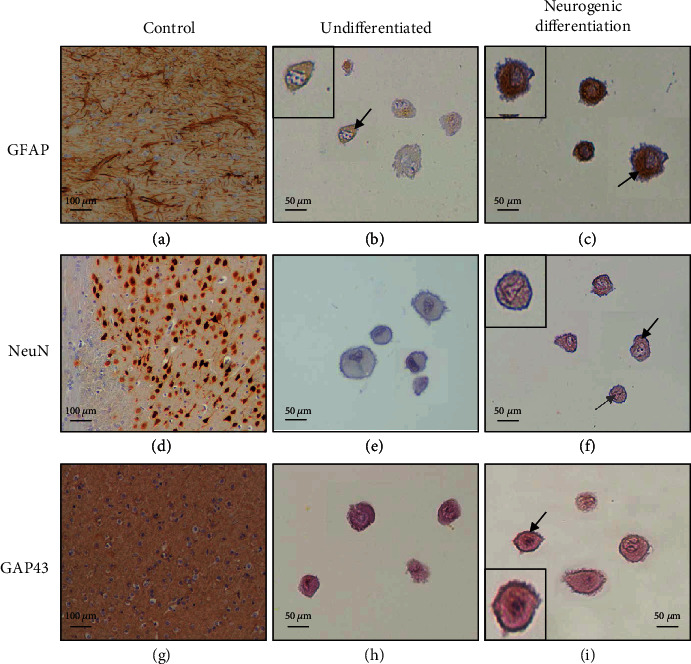
Immunolabeling of rat brain tissue (control) and of undifferentiated and differentiated OM-MSCs (P6). Magnifications: 100x (control) and 400x (OM-MSCs): (a) positive immunoexpression of the intermediate filaments of the astroglial cells; (b) (+) the black arrow highlights weak cytoplasm immunoreactivity; (c) (+++) the black arrow highlights strong cytoplasm immunoreactivity; (d) positive immunoexpression of neuronal nuclei; (e (0), f (+)) the black arrow highlights weak cytoplasm immunoreactivity; the grey arrow also highlights nuclear immunopositivity; (g) positive neuronal membrane and periplasmic immunoreactivity; (h (0), i (++)) the black arrow highlights moderate membranous immunoreactivity. The inserts highlight the immunoexpression of each marker.

The results of the immunohistochemistry evaluation can be analyzed in Figure 6. After neurogenic differentiation, OM-MSCs showed immunoreactivity for GFAP (+++), NeuN (+), and GAP43 (++). In undifferentiated cells, only immunoreactivity for GFAP (+) was observed.

### 3.3. *In Vitro* Cytocompatibility Assessment

**Figure 7:**
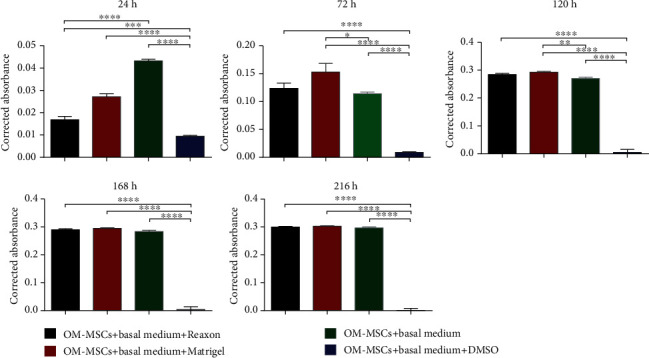
Corrected absorbance of the different groups assessed by the PrestoBlue® viability assay, in different time points. Results are presented as mean ± SEM.

The results of the cytocompatibility assay are described in Figure 7. The values obtained are in Table S2, and the statistical differences identified are described in Table S3.

At 24 h, OM-MSCs seeded only with basal medium (positive control) showed a clearly higher viability, with statistical differences with groups (*p* < 0.0001). The group supplemented with DMSO is, as expected, the one with the lowest viability in this time point, the position that it occupies in all time points until the end of the assay. At 72 hours, the groups in which OM-MSCs were seeded in contact with Reaxon® NGCs and on Matrigel® showed a metabolic activity superior to that of the positive control, with no statistical differences between these two groups, but with the group with Matrigel® showing statistical differences with the positive control (*p* = 0.0236). From this time point, the remaining three groups have very close viability values, and while at 120 h, there are still statistical differences between the positive control and the group with Matrigel® (*p* = 0.0021) and at 168 and 216 h, there are no longer differences. Overall, in the positive control, good viability was observed as early as 24 h, with a progressive increase over time; in the test groups, there seems to have been an initial delay in metabolic activity rate related to the presence of the biomaterial and Matrigel®, a delay that was quickly overcome with a progressive increase in viability over the different time points. For these three groups, the plateau seems to have been reached at 120 h, with reduced increments thereafter. As expected, supplementation with DMSO was shown to be harmful to the cells right from the initial time points.

### 3.4. Scanning Electron Microscopy Analysis

**Figure 8:**
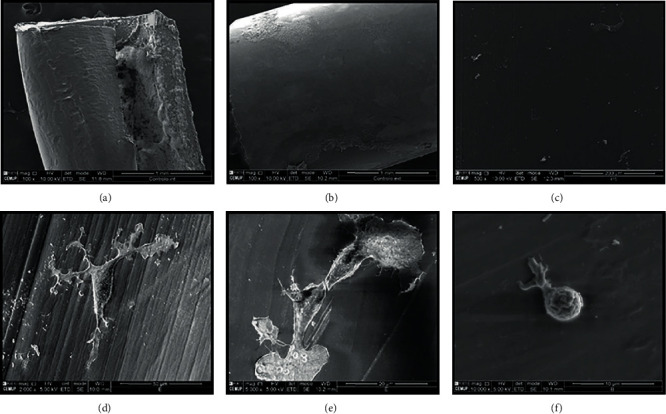
SEM images of the Reaxon® NGC and OM-MSCs: (a) Reaxon® NGC, magnification: 100x; (b) external surface of Reaxon® NGC, magnification: 100x; (c) inner surface of Reaxon® NGC, magnification: 500x; (d) OM-MSC adhered to the inner surface of the Reaxon® NGC, magnification: 2000x; (e) OM-MSCs adhered to the inner surface of the Reaxon® NGC, magnification: 5000x; (f) OM-MSC adhered to the inner surface of the Reaxon® NGC, magnification: 10000x.

**Figure 9:**
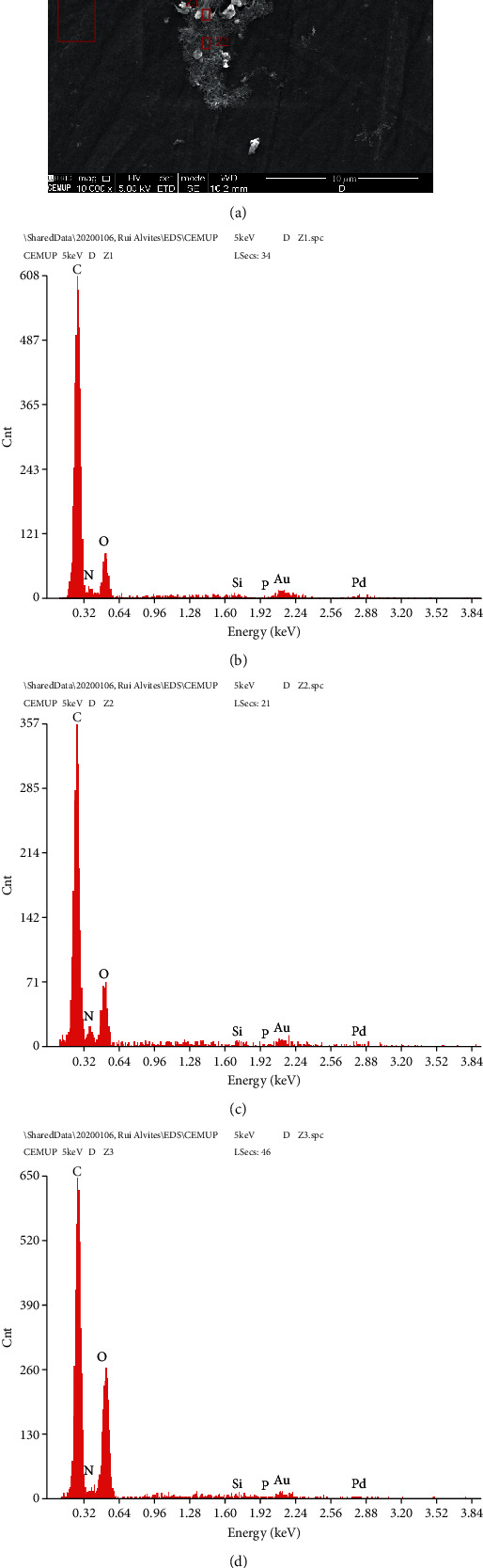
SEM and EDS evaluation of the Reaxon® NGC and OM-MSCs: (a) OM-MSC cell layer adhered to the inner face of the Reaxon® NGC, magnification: 10000x; (b) EDS evaluation of the Z1 region-OM-MSCs; (c) EDS evaluation of the Z2 region-OM-MSC cell layer; (d) EDS evaluation of the Z3 region-inner surface of the Reaxon® NGC without a cell layer.

The evaluation of the samples through SEM allowed observing the external and internal architecture of the Reaxon® NGCs (Figures 8(a)–8(c)), the morphology of OM-MSCs (Figures 8(d)–8(f)), and also the suitability of the biomaterial for cellular adhesion and proliferation, both individually and in homogeneous cell layers (Figure 9(a)). The EDS analysis allowed confirming the presence of cells and homogeneous cell layers, namely, through the identification of nitrogen (Figures 9(b)–9(d)).

### 3.5. Functional Assessment

#### 3.5.1. Motor Performance

**Figure 10:**
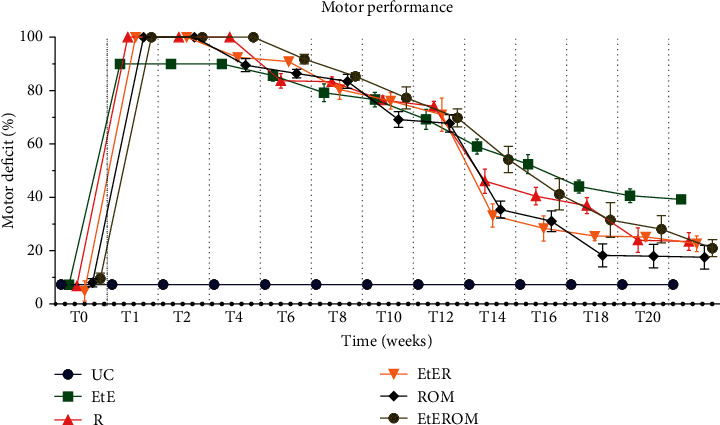
Values of motor deficit (%) over the 20 weeks of the recovery period. Results are presented as mean ± SEM.

The percentage of functional deficit (%) recorded until week 20 is shown in Figure 10. The complete values of functional deficit can be found in Table S4, and the statistical differences observed in T20 in Table S5.

After neurotmesis injury, a significant loss in hind limb strength and derived percentage of motor deficit were observed in all intervened groups. At week 1 postinjury, high deficit percentages were observed in all groups when compared to the UC group (*p* < 0.0001). Deficits gradually declined over the weeks, with variations in the position of the group with better results in each time point. From the fourteenth week onwards, a marked decrease in motor deficit was observed in almost all groups, except for the EtE group, where the variations up to the end of the study period were not so evident. After 20 weeks, the ROM group showed the lowest percentage of motor deficit, presenting statistically significant differences only with the EtE group (*p* < 0.0001). The EtE group was the one with the worst motor performance at the end of study time, showing statistically significant differences with all the other groups: R (*p* = 0.0045), EtER (*p* = 0.0024), and EtEROM (*p* = 0.0006). In either case, the motor deficit percentages were still severe in all intervened groups after 20 weeks, with statistically significant differences of all groups compared to the UC group (*p* = 0.0097 for ROM, *p* = 0.0006 for EtEROM, and *p* < 0.0001 for all other groups).

#### 3.5.2. Nociceptive Function

**Figure 11:**
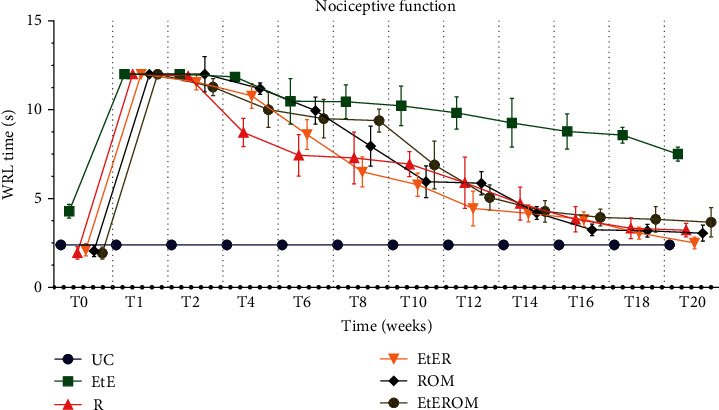
WRL values (s) over the 20-week recovery period. Results are presented as mean ± SEM.

The WRL values, in seconds, obtained over the 20 weeks are shown in Figure 11. The complete WRL values can be found in Table S6, and the statistical differences observed in T20 in Table S7.

After the neurotmesis injury, an increase in WRL time was observed for all the intervened groups. At week 1 postinjury, all experimental groups showed WRL times equal to the maximum value considered (12 seconds), with the need to remove the paw from contact with the heating plate to avoid thermal burns. At this point, no statistical differences were observed between the experimental groups, but between these and the UC group (*p* < 0.0001). From week 2 onwards, a decrease in WRL time began to be observed for all experimental groups. The EtE group had a much less pronounced decline in the WRL times, and in week 20, it ended up with a much higher value than the UC group (*p* < 0.0001) and other experimental groups (*p* < 0.0001). At 20 weeks, the group with the lowest WRL value was the EtER, but without significant statistical differences with the other experimental groups. In addition, excluding the EtE group, the remaining ones did not show statistical differences with the UC group.

#### 3.5.3. Walking Track Analysis

**Figure 12:**
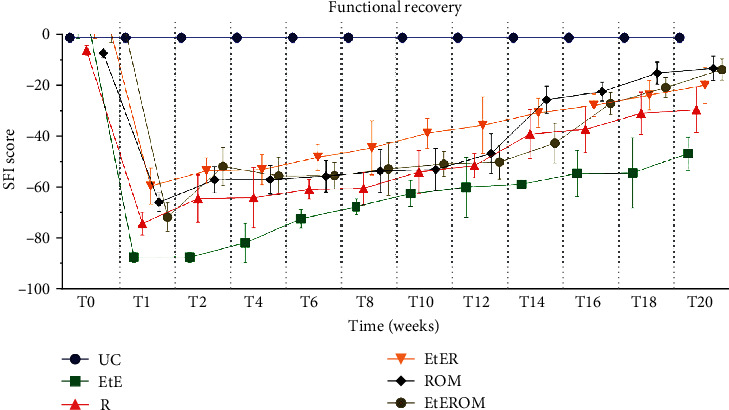
Functional assessment (SFI) over the 20-week recovery period. Results are presented as mean ± SEM.

The observed SFI values are shown in Figure 12. The complete SFI values can be found in Table S8, and the statistical differences observed in T20 in Table S9.

After the neurotmesis injury, a severe hind limb impairment was observed in all the intervened groups. In week 1 after the injury, despite the deficits observed in all groups, the EtE group showed the worst results, a position that remained until the end of the study period. At this point, statistically significant differences were observed between all therapeutic groups and the UC group (*p* < 0.0001) and also between the EtE and EtER (*p* = 0.0007), ROM (*p* = 0.0019), and EtEROM (*p* = 0.0003). From weeks 2-4, an increase in SFI values began to be observed, improvements that have remained consistently throughout the 20 weeks of study. In week 20, the ROM group recorded the best SFI value, but without statistical differences with the other therapeutic groups. The UC group showed statistical differences with the EtE (*p* < 0.0001), R (*p* = 0.0003), and EtER (*p* = 0.0349) but no statistical differences with the groups ROM and EtEROM.

**Figure 13:**
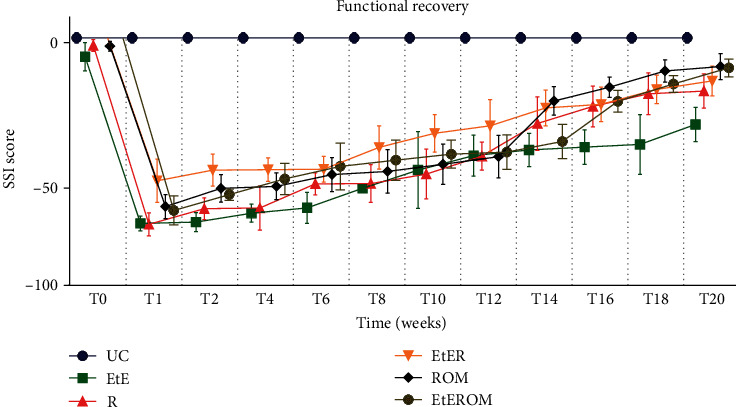
Functional assessment (SSI) over the 20-week recovery period. Results are presented as mean ± SEM.

The recorded SSI values are shown in Figure 13. The complete SFI values can be found in Table S10, and the statistical differences observed in T20 in Table S11.

Expectably, the graphical evolution regarding SSI is identical to SFI, confirming the effectiveness of the video and photographic recording method in capturing the performance of animals in the walking track analysis. At week 20, the EtE group is also the one with the worst functional result, with statistical differences with the ROM group (*p* = 0.0339). The ROM group was the one that presented the best result, only with statistical differences with the EtE group. The UC group showed statistical differences with EtE (*p* < 0.0001), R (*p* = 0.0058), and EtER (*p* = 0.0421) but no differences with EtEROM and ROM.

#### 3.5.4. Kinematic Analysis

**Figure 14:**
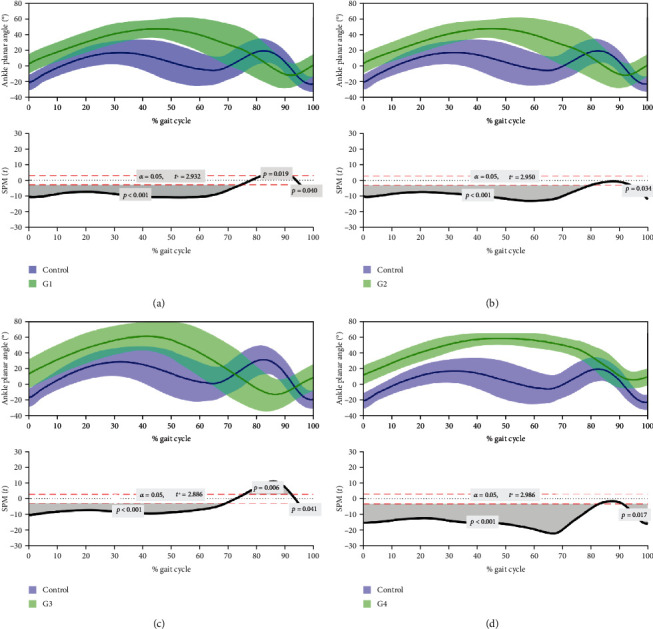
Kinematic evaluation of rats 20 weeks after neurotmesis: (a) R; (b) EtER; (c) ROM; (d) EtEROM. Upper graphs show the mean *θ*° during UC gait (blue) versus the pattern of experimental groups (green) at the level of the ankle joint. Lower graphs show SPM statistic as a function of the gait cycle. SPM unpaired *t*-tests were performed, comparing the mean kinematic angle of each pattern to the respective mean kinematic angle of UC (*α* = 0.05). The moments of the gait cycle in which the critical threshold (*t*^∗^) was exceeded (corresponding to the moment when the *θ*° of the experimental groups is superior to the *θ*° of UC) are represented by the grey area of the lower graphs. G1 = R; G2 = EtER; G3 = ROM; G4 = EtEROM; SPM: statistical parametric mapping.

The results of the kinematic evaluation are shown in Figure 14.

The gait pattern of the ankle joint in the sagittal plane significantly differed between the four intervened groups and UC. The 4 groups showed an increased *θ*° at least during the entire stance phase (*p* < 0.001 with UC). The main differences between therapeutic groups and UC waveforms were found in the swing phase. In the late swing phase, the 4 groups presented significant differences with the UC group (*p* = 0.013 with R, *p* = 0.011 with EtER, *p* = 0.013 with ROM, and *p* = 0.05 with EtEROM). Groups EtER and EtEROM presented no differences with the UC group during the middle swing phase (75-95% of the gait cycle), while groups R and ROM were significantly different from the UC group in this same time period (*p* = 0.006 and *p* = 0.002, respectively), revealing a marked plantarflexed joint position. Regarding the spatiotemporal parameters of the gait, all groups presented longer cycle times, with an increased stance phase (UC = 0.244 ± 0.125 ms, R = 0.871 ± 0.313 ms, EtER = 0.702 ± 0.320 ms, ROM = 0.395 ± 0.133 ms, and EtEROM = 0.506 ± 0.175 ms) and swing phase (UC = 0.131 ± 0.009 ms, R = 0.283 ± 0.036 ms, EtER = 0.245 ± 0.054 ms, ROM = 0.306 ± 0.046 ms, and EtEROM = 0.344 ± 0.070 ms).

Table 3 contains a qualitative representation of the performance of the therapeutic groups in the different essays and tests performed *in vivo*. In general, it is possible to see that the groups that received OM-MSCs and NGCs had a better overall performance, with good results in basically all parameters. However, the results are not regular, and in some tests, the R or EtER groups showed the same or better performances.

### 3.6. Stereological and Histomorphometric Analysis

#### 3.6.1. Nerve Stereological Analysis

**Figure 15:**
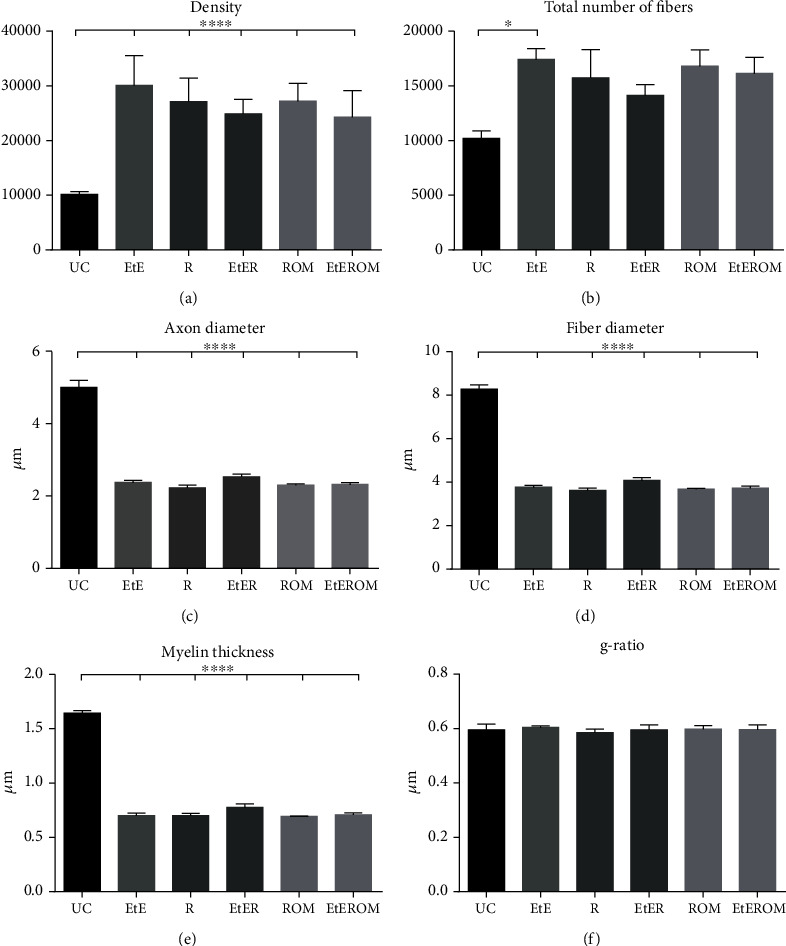
Results of the stereological assessment of sciatic nerve fibers 20 weeks after neurotmesis: (a) density; (b) total number of fibers; (c) axon diameter; (d) fiber diameter; (e) myelin thickness; (f) g-ratio. Results are presented as mean ± SEM.

**Figure 16:**
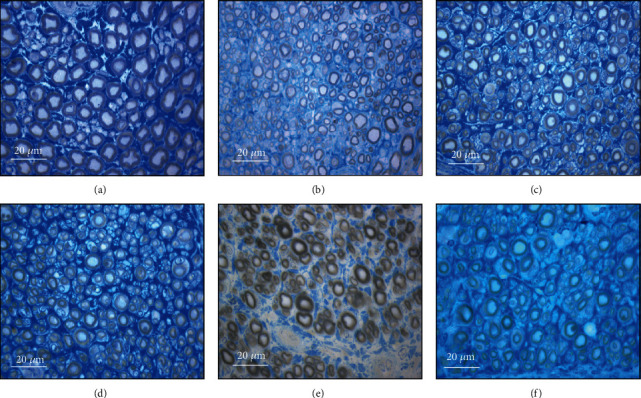
Light micrographs of toluidine blue-stained sciatic nerve semithin sections for the different groups: (a) UC; (b) EtE; (c) R; (d) EtER; (e) ROM; (f) EtEROM.

The results obtained in the stereological evaluation are shown in Figure 15. The corresponding stereological images can be seen in Figure 16. The total stereological results obtained can be found in Table S12, and the statistical differences observed are in Table S13.

All the therapeutic options considered demonstrated stereological evidence of nerve fiber regeneration. When compared to the control, after 20 weeks, the nerves submitted to different therapeutic options showed microfasciculation phenomena with axons and fibers of smaller diameter, higher density and number of nerve fibers, and thinner myelin sheaths. The therapeutic groups never showed statistical differences between them in any parameter. In the parameters density and number of fibers, the EtE group presented the highest value (30072 ± 5443 fibers/mm^2^ and 17423 ± 2217 fibers, respectively). In density and axon diameter, there are statistically significant differences between UC and all the other groups (*p* < 0.0001); in the total number of fibers, there are differences between UC and EtE (*p* = 0.0327).

The highest value of nerve fibers and axon diameter was observed in the EtER group (4.08 ± 0.33 *μ*m and 2.53 ± 0.18, respectively), and UC presents differences with all therapeutic groups (*p* < 0.0001). In the thickness of the myelin sheath, the highest value is that of the EtER group (0.78 ± 0.08 *μ*) and again UC has statistical differences with all other groups (*p* < 0.0001). The largest cross-sectional area was reached in the EtEROM group (0.6917 ± 0.2441 mm^2^), and UC presented statistical differences with all groups (*p* = 0.0337 with EtE, *p* = 0.0331 with R, *p* = 0.0105 with EtER, and *p* = 0.0436 with ROM) except EtEROM (data not shown). Regarding the g-ratio, the group with the highest value was the EtE (0.60 ± 0.01).

#### 3.6.2. Histomorphometric Muscle Analysis

All cranial tibial muscles associated with sciatic nerve injury had lower final weights than the corresponding contralateral muscles at the time of collection, with an average loss of 35.05 ± 4.69%. The highest percentage of loss was observed in group R (42.57 ± 10.60%), and the smallest in the ROM group (30.94 ± 14.07%).

**Figure 17:**
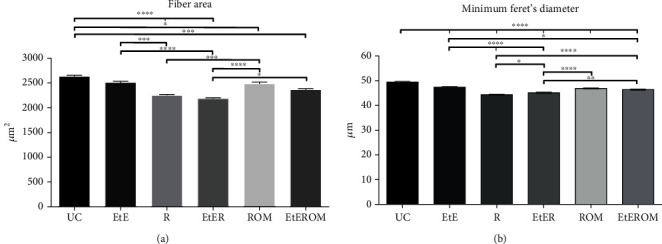
Histomorphometric analysis of cranial tibial muscle: (a) individual fiber area; (b) minimum Feret's diameter of the muscle fibers. Results are presented as mean ± SEM.

**Figure 18:**
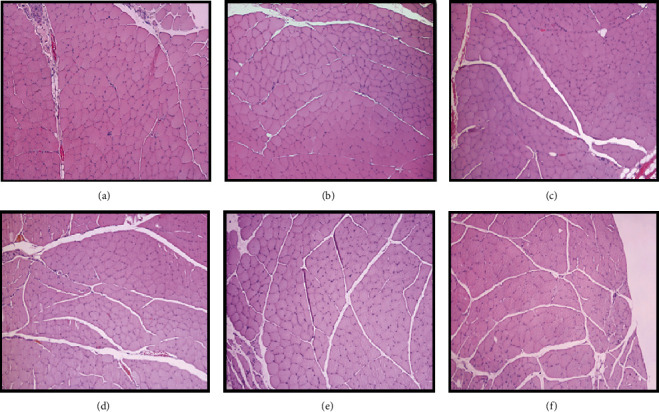
Histological images of the cranial tibial muscles subjected to histomorphometric analysis in the different groups: (a) UC; (b) EtE; (c) R; (d) EtER; (e) ROM; (f) EtEROM. H&E, magnifications: 100x.

The values of the fiber area and minimum Feret's diameter resulting from the evaluation of the cranial tibial muscles are shown in Figures 17(a) and 17(b), and the histological images of the different groups can be seen in Figure 18. The statistical differences observed are found in Table S14.

Regarding the fiber area, the therapeutic groups with the best results were EtE (2510.91 ± 23.08 *μ*m^2^) and ROM (2483.38 ± 35.91 *μ*m^2^). The EtE group did not show statistically significant differences with the UC group (2634.79 ± 24.01 *μ*m^2^), unlike ROM (*p* = 0.0438). The EtE group showed statistical differences with the groups R (*p* = 0.0003) and EtER (*p* < 0.0001), as well as ROM (R (*p* = 0.0004) and EtER (*p* < 0.0001)). The worst result was observed in the EtER group (2182.484 ± 18.41 *μ*m^2^), which only shows no statistically significant differences with the R group (2250.50 ± 18.57 *μ*m^2^), the second with the worst outcome (*p* = 0.0438 with EtEROM). The R group did not show statistical differences with the EtEROM group (2366.69 + 1992 *μ*m^2^). Excluding the EtE group, the UC group presented statistically significant differences with all other therapeutic groups (R, EtER (*p* < 0.0001); EtEROM (*p* = 0.0010)).

In the case of the minimum Feret's angle, the best values were also observed in the EtE (47.42 ± 0.23 *μ*m) and ROM (46.87 ± 0.23 *μ*m) groups, but in this case, the UC (49.43 ± 0.25 *μ*m) group showed statistically significant differences with all therapeutic groups (*p* < 0.0001). The EtE group did not differ statistically from ROM, unlike with other groups (R and EtER (*p* < 0.0001); EtEROM (*p* = 0.0154)); The ROM group differs statistically from the R and EtER groups (*p* < 0.0001). The worst outcome was observed in group R (44.37 ± 0.15 *μ*m), followed by EtER (45.15 ± 0.22 *μ*m) with w5hich it has statistical differences (*p* = 0.0272). The EtEROM (46.38 ± 0.25 *μ*m) group also presents statistically significant differences with the two worst groups, R (*p* < 0.0001) and EtER (*p* = 0.0098).

## 4. Discussion

The success resulting from the repair of a peripheral nerve after PNI depends on the type and extent of the injury. In neurotmesis injuries, the most severe, the complete transection of the nerve culminates in the loss of continuity and function, axonal and structural disorganization, and loss of the coating elements and myelin sheath [78]. The use of NGCs is a valid alternative to traditional techniques for treating neurotmesis injuries such as EtE sutures and autographs, ensuring the maintenance of a proregenerative environment within it and less influenced by the organic immune response that has, sometimes, detrimental effects [79]. These NGCs can consist of materials with natural and artificial origin, and their chemical and physical characteristics can be modified and adapted to ensure the best performance in promoting nerve regeneration and controlling inflammatory reaction [80]. However, despite the various materials proposed so far, none yet guarantee an optimal functional recovery after conduit application. The current most promising options include modifying the NGCs' inner architecture, transplanting MSCS to its lumen, and also including ECM and neurotrophic factors in the therapeutic approach. In fact, the nerve regeneration promoted in this work, using a combination of chitosan NGCs and OM-MSCs suspended in Matrigel®, allowed observing good functional, kinematic, and histomorphometric outcomes in animals subjected to neurotmesis, although the results are not yet good enough to equate to complete regeneration.

Chitosan has been used in medical applications for a long time due to its biodegradability, biocompatibility, and absence of toxicity. Although this biomaterial has been applied in various forms and with different surgical techniques, the number of works where it has been used as NGCs is reduced, particularly as NGCs consisting solely of chitosan [16]. One work that studied the benefits of applying Reaxon® NGCs in the primary repair of peripheral sensory nerves demonstrated better outcomes of tactile gnosis and sensitivity than the traditional EtE suture technique [21]. Another work explored the application of fresh skeletal muscle fibers in combination with Reaxon® conduits to promote the regeneration of the rat median nerve after neurotmesis. However, although this combined technique seems to have advantages, namely, with an increased production of neuregulin, the observation of no statistical differences with the remaining therapeutic groups in terms of functional recovery and morphometric analyses did not allow drawing any broader conclusions [81]. In this work, Reaxon® NGCs with 2.1 mm internal diameter were used in different surgical techniques to stimulate rat sciatic nerve regeneration after neurotmesis, both alone and in combination with OM-MSCs and Matrigel®.

OM-MSCs have only recently begun to be explored in regenerative medicine and applied to the nervous system. In a study that compared the use of biphasic collagen and laminin-functionalized hyaluronic acid NGCs with and without supplementation with OM-MSCs, the combined technique revealed better clinical and electrophysiological outcomes than the use of NGCs alone, with a greater number of axons and a more rapid return of nociceptive withdrawal reflex to normal values [45]. The use of rat OM-MSCs in this work follows a wide study of characterization of these cells [23]. A complete biological characterization was carried out, with identification of karyotype normality in different passages, capacity for tridifferentiation and neurogenic differentiation, and confirmation of their stemness characteristics, allowing the establishment of these cells as adequate to be applied therapeutically in regenerative medicine. In addition, it also allowed identifying several genes characteristic of MSCs and respective differentiations and also specific surface markers. A preliminary characterization of the conditioned medium of the OM-MSCs at 48 h also allowed identifying immunomodulatory and immunosuppressive factors, chemokines, growth factors, and interleukins important in regenerative phenomena, complementing findings identified by other authors [82]. Since in this previous study the genes and surface markers associated with the potential for neurogenic differentiation were identified in OM-MSCs in their undifferentiated state and the capacity for neurogenic differentiation was tested exclusively by observing the acquisition of neuroglial-like shape in culture, in the present study, an additional biological characterization was carried out to identify genes and surface markers related to neurogenic differentiation, comparing their presence and expression between undifferentiated cells and after neurogenic differentiation. Prior to its *in vivo* application, the cytocompatibility between OM-MSCs and Reaxon® and between cells and Matrigel® was tested, to ensure that cell viability and metabolism would not be adversely affected by the contact between cells and the biomaterial and ECM substitute. Additionally, and complementarily, Reaxon® tubes that received seeded OM-MSCs were analyzed by SEM and EDS to confirm the adhesion of these cells to their internal surface without morphological changes and with the formation of homogeneous cell layers. After these preliminary tests, finally the chitosan NGCs combined with OM-MSCs and Matrigel® were used in the sciatic nerve of rats after neurotmesis lesions, testing the capacity of this therapeutic approach to stimulate peripheral nerve regeneration after PNI. This capacity was tested *in vivo* by functional and kinematic studies and *postmortem* through histomorphometric and stereological evaluation.

With the RT-PCR technique, 22 genes associated with neurogenic differentiation were quantified in undifferentiated and differentiated OM-MSCs. It was also possible to identify changes in the fold expression of these genes between cells of the two groups. In both groups, cells in P6 were used, according to the increased propensity of the cells to differentiate in this passage, as previously identified [23]. The absence of contamination in the RNA sample used for cDNA synthesis was confirmed by spectrophotometry, and the interpretation of the melting curves confirmed the presence of a single peak, that is, amplification of a single amplicon for each gene. The identification of genes characteristic of neuroglial cells in undifferentiated cells must be interpreted as a propensity for these cells to follow neurogenic differentiation when subjected to the appropriate prodifferentiation stimuli. In the differentiated cells, the presence of genes can already be considered having an active function in their specific functions and acquired phenotypes. The MPZ gene, expressed specifically in the Schwann cells of the peripheral nervous system and responsible for a transmembrane glycoprotein with structural function in the peripheral myelin sheath [83], was the only one not identified in any of the cell groups. The Dcx (microtubule-associated phosphoprotein expressed in immature postmitotic neurons, required for neuronal migration [84]), Rbfox3 (specific protein from mature postmitotic neurons [85]), and Syp (regulation of vesicular endocytosis during the synapse of mature neurons [86]) genes were identified in the differentiated cells but not in the undifferentiated ones, which may indicate a differentiation of OM-MSCs in a neuron-like direction. It is important to note that synaptophysin has been previously identified in undifferentiated OM-MSCs through immunohistochemistry techniques, although with weak immunoreactivity [23]. Ocln (expressed in neuroepithelial cells in precocious stages of neurogenesis [87]) was only expressed in the undifferentiated cells, being absent as expected in the differentiated cells. Olig3, expressed in mature oligodendrocytes [88], was also only expressed in undifferentiated cells. All the remaining genes were identified in the two groups of cells, in no case with downregulation in the differentiated cells. The genes with the highest RQ value were Aldh1l1 (astrocytic differentiation marker [89]) and Cd40 (expressed in microglia [90]), that is, genes associated with glial cells in the central nervous system. Other genes associated with glial cells upregulated in differentiated OM-MSCs include Cdh2 (expressed in radial glia [91]), GFAP (fibrillary protein characteristic of glial cells expressed in astrocytes and radial glia [92]), Ncam1 (glycoprotein related to cell adhesion in nonmyelinating Schwann cells [93]), Nes (intermediate filament protein expressed in the undifferentiated central nervous system, in radial glia before the development of astrocytes, but also in the mature central nervous system [94]), Sox2 (persistent marker for multipotential neural stem cells expressed in proliferating cells and those that follow glial differentiation, but not expressed in postmitotic neurons [95]), and Sox10 (present in neuroepithelial cells and myelinating Schwann cell precursors [96]). GFAP and Sox2 have also been previously identified in undifferentiated OM-MSCs [23]. In turn, genes associated with neurons, upregulated, include Ascl1 (promoting neural differentiation, namely, in the generation of olfactory neurons [97]), Cdksr1 (different functions from neuronal differentiation and migration to synaptic transmission [98]), Cript (postsynaptic protein in excitatory synapses [99]), Map2 (microtubule stabilizer in the dendrites of postmitotic neurons [100]), and Tubb3 (immature neuron-specific tubulin also involved in peripheral axon regeneration [101]. Finally, the Cspg4 (associated with myelinating oligodendrocyte precursors [102]), GAP43 (common marker found in neurons undergoing differentiation, being highly expressed in neurons in development or in regeneration during axonal growth, also expressed in nonmyelinating Schwann cells [103, 104]), and Neurod1 (associated with immature neurons, promoting neural development, and inducing terminal neuronal differentiation in olfactory neurogenesis [105]), although expressed in both groups of cells, are neither up- nor downregulated in the differentiated OM-MSCs. In general, the results of RT-PCR show that when undergoing neurogenic differentiation, OM-MSCs not only acquire a neuroglial cell phenotype but also present significant upregulation of neuroglial genes.

To complement the results obtained in the RT-PCR, an immunohistochemical evaluation was performed to identify the GFAP, NeuN (Rbfox3), and GAP43 protein immunomarkers. The GFAP marker was identified in both undifferentiated and differentiated cells, but with weak and strong immunoreactivity in the first group and after neurodifferentiation, respectively. This result corroborates that obtained in the RT-PCR in which upregulation of GFAP was detected among the differentiated cells. GFAP had previously been identified in undifferentiated OM-MSCs [23]. NeuN expression was identified only in neurodifferentiated cells with weak immunoreactivity, and in RT-PCR, Rbfox3 expression was also only observed in cells after differentiation. GAP43 was expressed only on the membrane of differentiated cells, with moderate reactivity. In RT-PCR, although GAP43 was identified in both groups of cells, neither upregulation nor downregulation was observed after neurodifferentiation.

The RT-PCR and immunohistochemistry assays followed the same line of results, demonstrating an overexpression of neuroglial immunomarkers in cells after neurogenic differentiation of cells. However, since upregulation and immunoreactivity of both neuron-related and glia-associated genes and protein markers were noticed, it is difficult to define whether there is a predominant tendency for OM-MSCs to differentiate into some specific neuroglial cell type. Probably, *in vivo*, OM-MSCs will follow different lines of neural or glial differentiation depending on the implantation site and the local stimuli that they receive.

The application of biomaterials in the human body or in animal models must obligatorily follow certain requirements: being biologically safe (without toxic effects and without generating immune reactions in the host), biofunctional, and resistant to degradation (excluding biodegradation intrinsic to the material). These characteristics can be confirmed by determining their biocompatibility [106]. The term cytocompatibility, that is, the *in vitro* behavior and metabolic performance of specific cells when seeded in contact with a material, can be considered a particular case of biocompatibility, which is the wider term [107]. The cytocompatibility between OM-MSCs and Reaxon® and Matrigel® was studied using a PrestoBlue® cell viability assay. PrestoBlue® is a blue reagent based on resazurin, for which cell membranes are permeable, which is metabolized at the mitochondria, resulting in resorufin, a red fluorescent compound that can be measured both visually and through its absorbance, establishing a direct relationship with the viability and metabolic activity of the studied cells [108]. The results of this assay revealed that the presence of chitosan conduits and Matrigel® temporarily delayed cell adhesion, with differences in viability and metabolic performance being observed at 24 h compared to OM-MSCs that were seeded only with the usual culture medium. However, from this point on, the viability associated with the biomaterial and Matrigel® increased, and for later time points, there were no differences between the positive control and the test groups. To confirm the microscopic interaction between OM-MSCs and Reaxon® conduits, the chitosan tubes on which the cells were seeded were evaluated by SEM and EDS, an evaluation in which it was possible to confirm the permissible characteristics of Reaxon®. OM-MSCs adhered to the internal surface of the conduits, maintaining their normal morphology and forming cell layers. The EDS technique, when used in combination with SEM, allows the identification of the elements present near the sample surface to be analyzed, creating a graphic map of the chemical components identified through the energy of the emitted X-rays [109]. In this case, in all the analyzed regions, it is possible to see the predominance of the elements carbon and oxygen, due to the chemical constitution of chitosan. However, the evaluation of the Z1 and Z2 regions allows the identification of nitrogen peaks, which, being a fundamental cellular chemical component, works here as an identifier for the presence of cell material adhering to the surfaces of the NGC, confirming that the structures identified in the SEM are, in fact, OM-MSCs and cell layers adhering to the internal surface of the chitosan NGC.

After the additional biological characterization of OM-MSCs and the determination of the cytocompatibility between MSCs, Reaxon®, and Matrigel®, the efficacy of the chosen therapeutic combinations *in vivo* was evaluated. After surgical application of the five therapeutic options, the animals were regularly evaluated over 20 weeks to determine the progression of motor, nociceptive, and behavioral performance and gait characteristics. At the end of the study period, the animals were subjected to a kinematic evaluation and the sciatic nerves and cranial tibial muscles were collected for stereological and histomorphometric evaluation, respectively.

The percentage of motor deficit decreased significantly in all animals during the first weeks after neurotmesis. Between the sixth and eighth week, an improvement in motor performance began to be observed in all groups, but it is from the fourteenth week that this improvement becomes more evident, indicating superior motor nerve fiber regeneration. At 20 weeks, all groups had a percentage of motor deficits much lower than the postsurgery values, revealing the efficacy of all therapeutic approaches in promoting the recovery of motor function. The ROM group was the one with the best results. With the exception of the EtE group, the one with the worst outcome, there were no statistical differences between the groups under test, but in none of them, a motor performance similar to that of the control group was achieved.

The WRL also increased to the maximum value considered after neurotmesis (12 s), indicating the total absence of thermal sensitivity in this phase. Between the second and the fourth week after surgery, a decrease in WRL time was observed, indicating a regeneration of sensory nerve fibers. This improvement, less marked in the EtE group, continued until the end of 20 weeks. The best outcome was observed in the EtER group, but the values of all study groups were low enough to not observe statistical differences between them and the UC group. These good results in nociceptive assessment corroborate those observed in other studies using Reaxon® NGCs [21] and OM-MSCs [45].

The observed results of SSI and SFI are concordant. In both cases, after neurotmesis, the values fell close to the minimum values considered after injury. Between the second and the fourth week, there was an improvement in the values of both indexes, values that increased continuously until the end of the 20 weeks. The EtE group was, again, the one with the worst outcome, and the ROM and EtEROM groups were the ones with the best results, without statistical differences with the UC group. Only the groups that did not receive OM-MSCs and Reaxon® showed statistical differences with UC. The videographic and photographic recording technique used in this work also made it possible to replace the traditional technique of recording footprints with ink, making the process faster and easier to perform and the results easier to interpret.

Overall, it is possible to state that all the therapeutic groups promoted improvements in the functional parameters evaluated, with the groups receiving OM-MSCs, Reaxon®, and Matrigel® promoting the best final results in motor performance and walking tract analysis and the EtER group promoting the best nociceptive recovery. The nociception and sciatic index final values were good enough to avoid statistical differences between groups with better outcomes and control groups, the fact that was not registered in the motor recovery. The EtE group showed the worst performance in all tests.

Regarding the kinematic results, an alternative statistical strategy was applied using global variables for curve alignment, reducing individual joint data to overcome the joint kinematic interanimal variability after peripheral nerve lesions, as proposed in previous works [110–112]. It was possible to measure the movement deficits of the ankle joint after PNI and verify the severity during the swing phase of walking. It is during the swing phase, instead of during the stance phase, that motion dysfunction is more evident after sciatic nerve denervation [112]. EtER and EtEROM revealed a similar pattern in terms of gait deviations when compared with the UC group, especially during the swing phase, with the EtEROM group presenting the best performance among the therapeutic groups. These two groups have in common the fact that both sciatic nerves have been subjected to an EtE suture associated with therapeutic intervention with NGC and OM-MSCs, but considering the poor results observed in the EtE group in the functional assessment and also the results of the kinematic evaluation obtained in previous studies [113], the influence of this traditional suture on this good kinematic performance should be interpreted with caution. The ROM group was the one that showed the worst results in the kinematic evaluation, an unexpected result considering the good performance of this group in almost all other tests. Ankle motion deficits affecting the swing phase demonstrate the lack of ability of intervened animals to actively perform dorsiflexion and plantarflexion. A decreased ankle dorsiflexion angle at the midswing, defined as the instant where the swinging leg crosses the contralateral hind limb, has already been reported after sciatic nerve axonotmesis [112, 114, 115], and it is also observed in this case, even in animals belonging to groups with better recovery and kinematic performance. Although the contractures observed in most animals immediately after the neurotmesis lesion are attenuated over time, functional recovery is not complete, and after twenty weeks, there are still kinematic indications of dysfunctions in gait and its components/phases. Consequently, the duration of the stance phase and the swing phase is longer than the times considered standard for the rat [116].

The analysis and quantitative estimation of nerve fiber morphology are an essential assessment in determining the degree of nerve regeneration after injury and treatment, and stereology should always be interpreted in combination with functional and kinematic results [75]. In this work, all nerves that suffered neurotmesis and had been subjected to specific therapies presented, after 20 weeks, microfasciculation, high density and number of nerve fibers, axons with small diameter, and thin myelin sheaths, characteristics normally observed in nerves that have undergone a regeneration process. The corresponding images in Figure 16 corroborate the stereological findings. The results obtained in this analysis must, however, be evaluated with extra care. The number and density of nerve fibers are often interpreted as direct indicators of nerve regeneration. A high number of regenerated nerve fibers can be associated not only with a good regenerative process but also with changes such as aberrant sprouting, and a high density can also reflect the presence of a large number of axons, but of small dimensions. Conversely, a lower fiber density may represent the presence of larger axons but also the presence of intraneural edema. Usually, shortly after the nerve injury, the tendency is to see an increase in the number and density of nerve fibers and axons of smaller diameter during nerve regeneration, a situation that is reversed over time, when the axons tend to increase their diameter and the number and density may decrease [117]. The EtE group was the one that presented higher values of density and number of fibers, being therefore, and considering the results of the functional assessment, the one that is in a less advanced phase of nerve regeneration after twenty weeks. EtEROM and EtER had the lowest values of density, and EtER the lowest number of fibers. The diameter of the nerve fiber is the main determinant of conduction velocity since it informs about the axonal diameter and the thickness of the myelin sheath [118]. For the three parameters, *d*, *D*, and *M*, the EtER group is the one that presents the best stereological performance, but the fact that its values are very different from those observed in the UC group and that there are no statistical differences between the therapeutic groups does not allow more extended conclusions. The g-ratio relates the axonal diameter to the total diameter of the nerve fiber (*d*/*D*) and is commonly analyzed as a functional and structural index that reflects the quality of the axonal myelination. Traditionally, the value of 0.6 was established as the optimal g-ratio for peripheral nerves [119, 120]. Consequently, deviations in the thickness of the myelin sheaths, and therefore g-ratios bigger or smaller than 0.6, promote a decrease in conduction speeds [121]. The g-ratio observed in this work reveal values of 0.6 or very close for all groups (UC and therapeutic groups) and, therefore, with no statistical differences observed. This result seems to indicate that, despite the stereological differences observed in the therapeutic groups, the interventional nerves developed good axonal myelination and potential for efficient conduction speeds, which corroborates the results observed in the remaining performed tests. Overall, the stereological assessment of the intervened nerves reveals a stimulation of nerve regeneration and identical performances among all the therapeutic groups tested. However, the absence of statistical differences between the groups considered and the fact that the final values fall below those observed in the control group for almost all parameters do not allow establishing any therapeutic option as the most advantageous.

Histomorphometric assessment of the anterior tibial muscle, as an effector muscle of the sciatic nerve, revealed different neurogenic muscle atrophies depending on the therapeutic approach. The best final values of the fiber area and minimum Feret's angle were observed in the EtE group, in the first case without statistical differences with the control group, results that deviate from the poor performance of this surgical approach in the remaining evaluations. In both cases, the ROM group was the second with the best muscle parameters and was also the one where the percentage of muscle mass loss was lower, corroborating the good performance of this therapeutic option in the functional evaluation. The groups where the biomaterial was used alone were those with the worst results. These outcomes seem to indicate that in the EtE group, there was an earlier reestablishment of the nerve connections, perhaps due to possible factors that temporarily delay cell activity in other groups, such as t5he presence of biomaterial and Matrigel® as demonstrated in cytocompatibility assays, resulting in an also earlier nervous reestimulation of the anterior tibial muscle and avoiding/decreasing neurogenic atrophy. Despite this early reconnection, functional recovery in this group was not as effective as in the remaining therapeutic groups, and the EtE group showed the worst outcomes than the other groups in terms of motor and nociceptive recovery and sciatic indexes. At the end of 20 weeks, the ROM and EtEROM groups demonstrate a performance similar to EtE, which may indicate an early reconnection associated with the use of OM-MSCs that does not seem to have happened in the groups that only received Reaxon®. It is interesting to note that ROM and EtEROM groups also showed the best results in the EPT, SFI, and SSI tests, which confirms the importance of avoiding/reversing neurogenic muscle atrophy to maintain a good motor performance and guarantee the return to a normal behavioral activity during gait.

Excluding in the histomorphometric evaluation of the cranial tibial muscle, the EtE group presented a poor performance in all essays, which can be considered unexpected since this technique is still considered, together with autografting, gold standard in the treatment of neurotmesis when it is possible to obtain a tension-free coaptation and a well-vascularized bed at the injury site. However, the observation of weaker therapeutic performance in cases treated with EtE than in those that received tubulation repair is not exclusive [122, 123]. Despite the juxtaposition and oriented alignment of the two nerve tops before the EtE suture, it is very difficult to guarantee an accurate axon-to-axon or endoneurium-to-endoneurium perfect alignment and reconnection, which can often translate into phenomena of misdirection and establishment of aberrant motor/sensory connections in the regenerating nerve. On the other hand, it has already been proven that the use of NGCs manages to mitigate the phenomena of misdirection [124], and when used in combination with MSCs, they are capable of promoting a proregenerative environment, prolonged over time, which translates into a better performance of these therapies in relation to the traditional application of sutures.

Despite the promising results obtained, it is important to consider some limitations that must be overcome in future works. The small number of animals used in each group can limit the extent of the conclusions reached, and although the rat model is a good starting point, to explore the therapies studied here in a translational perspective, it is important to consider for future essay the use of models with greater complexity that better mimic the speed and characteristics of human nerve regeneration, which differs significantly from that observed in the rat model [125]. In addition, it will also be important to carry out other functional assessment tests that consider a dimension that was not explored here, namely, nervous and muscular electrophysiological essays that will allow obtaining important information about the restitution of the nerve conductivity after regeneration and that were not evaluated in this work.

## 5. Conclusions and Further Directions

The combined use of MSCs and biomaterials to promote peripheral nerve regeneration after PNI is an increasingly applied therapeutic technique with promising results. OM-MSCs, despite the few published works, seem to be a type of MSCs with desirable characteristics to be applied in nerve regeneration, and the use of chitosan NGCs has proven to be an effective therapy. In this work, the initial biological characterization of OM-MSCs allowed confirming through PCR and immunohistochemistry techniques the tendency of these cells to follow a neurogenic differentiation, not only morphologically but also through the expression of neuroglial markers. Additionally, both cytocompatibility studies and its use in different therapeutic groups did not lead to local reactions or evidence of systemic toxicity associated with the presence of OM-MSCs, which reinforces the safety in the therapeutic use of these cells.

The functional assessment, kinematic analysis, and histomorphometric evaluations confirmed that the use of Reaxon® NGCs and OM-MSCs promoted functional improvements throughout the study period and evidence of nerve regeneration after 20 weeks. The best motor performances and sciatic indexes were observed in the groups that received both NGCs and OM-MSCs, ROM and EtEROM, and the nociceptive function recovered more effectively in the EtER group. Gait dysfunctions after twenty weeks were less evident in the EtEROM group. All therapeutic groups had identical stereological results, although the EtER group showed slightly higher values in important parameters such as axonal diameter, nerve fiber diameter, and myelin sheath thickness. The EtE technique obtained the best results in the histomorphometric evaluation of the cranial tibial muscle, but with the ROM and EtEROM groups showing identical performances.

In conclusion, the results obtained in this work demonstrate promising effects of the use of OM-MSCs and Reaxon® NGCs in promoting the regeneration of injured peripheral nerves and may open new therapeutic paths. However, there were also irregularities in some results of the different therapeutic groups, and in most of the essays, the results obtained are below those observed in the control group. This scenario reinforces the fact that nerve regeneration is a complex and multifactorial process, and to fully understand the regenerative efficacy of the treatments instituted, an integrated approach is always necessary, using different functional and histomorphometric assessment techniques that evaluate both dimensions associated with nerve regeneration: structure and function. Further studies are necessary to answer the doubts raised and establish more reasoned therapeutic approaches using OM-MSCs and biomaterials.

## Figures and Tables

**Table 1:** List of antibodies investigated, dilutions, and antigen retrieval methods applied in the immunohistochemical analysis.

Antibody	Clonality	Manufacturer	Dilution	Antigen retrieval
GFAP	Polyclonal	Proteintech®	1/1000	Water bath/20 min
NeuN	Polyclonal	Proteintech®	1/800	Water bath/20 min
GAP43	Polyclonal	Proteintech®	1/500	Water bath/20 min

**Table 2:** Ct, ΔCt, ΔΔCt, and RQ values for all genes under study for OM-MSCs undifferentiated and after neurogenic differentiation. N = normal; *↑* = upregulated; ↓ = downregulated; nd = nondefined.

Target gene	Undifferentiated		Differentiated				
	Ct average	ΔCt	Ct average	ΔCt	ΔΔCt	RQ	Regulation
Aldh1l1	36.5 ± 0.4	13.1	32.8 ± 0.3	7.4	-5.7	51.9	↑
Ascl1	38.6 ± 1.8	15.2	39.5 ± 0.7	14.1	-1.1	2.2	↑
Cd40	38.7 ± 0.7	15.4	35.1 ± 1.0	9.6	-5.7	53.2	↑
Cdh2	30.1 ± 0.4	6.8	30.1 ± 0.6	4.7	-2.1	4.2	↑
Cdk5r1	36.4 ± 0.8	13.0	36.3 ± 1.3	10.9	-2.2	4.5	↑
Cript	26.9 ± 0.2	3.5	26.5 ± 0.4	1.0	-2.5	5.6	↑
Cspg4	28.5 ± 0.3	5.1	29.9 ± 0.2	4.4	-0.7	1.6	*Ν*
Dcx	nd	nd	39.1 ± 0.0	13.6	nd	nd	nd
Gap43	37.7 ± 1.1	14.3	39.1 ± 1.9	13.6	-0.7	1.6	N
Gfap	36.3 ± 0.8	12.9	36.9 ± 0.3	11.5	-1.4	2.6	↑
Map2	31.5 ± 0.2	8.1	30.5 ± 0.4	5.1	-3.0	8.2	↑
Mpz	nd	nd	nd	nd	nd	nd	nd
Ncam	34.9 ± 0.9	11.4	35.6 ± 2.0	10.2	-1.2	2.3	↑
Nes	33.0 ± 0.2	9.6	33.6 ± 0.4	8.2	-1.4	2.7	↑
Neurod1	36.3 ± 0.2	12.9	38.4 ± 1.9	12.9	0.0	0.1	N
Ocln	37.6 ± 1.2	14.3	nd	nd	nd	nd	nd
Olig3	40.7 ± 0.0	17.3	nd	nd	nd	nd	nd
Rbfox3	nd	nd	37.7 ± 0.0	12.3	nd	nd	nd
Sox10	36.8 ± 1.6	13.4	37.2 ± 1.8	11.8	-1.6	3.0	↑
Sox2	36.3 ± 0.0	12.9	36.1 ± 0.0	10.7	-2.2	4.7	↑
Syp	nd	nd	36.4 ± 1.2	11.0	nd	nd	nd
Tubb3	29.8 ± 0.5	6.4	29.4 ± 0.7	4.0	-2.4	5.4	↑

**Table 3:** Qualitative general classification of the performance of the therapeutic groups in the tests and essays carried out *in vivo*. The groups were classified according to the statistical differences observed between them and the UC group: ∗∗∗∗ (-); ∗∗∗ (±); ∗∗, ∗ (+); and no statistical differences (++).

		EtE	R	EtER	ROM	EtEROM
Functional assessment	EPT	-	-	-	+	±
	WRL	-	++	++	++	++
	SFI	-	±	+	++	++
	SSI	-	+	+	++	++
	Kinematics		±	++	±	++

## Data Availability

The data that support the findings of this study are available from the corresponding author on request.

## Abbreviations

*θ*°: Ankle angle
Ct: Threshold cycle
DMSO: Dimethyl sulfoxide
ECM: Extracellular matrix
EDS: Energy-dispersive X-ray spectroscopy
EEPT: Experimental extended postural thrust
EPT: Extended postural thrust
EtE: End-to-end suture
EtER: EtE and wrapping with Reaxon® NGC
EtEROM: EtE, wrapping with Reaxon® NGC, and administration of OM-MSCs suspended in Matrigel®
HMDS: Hexamethyldisilazane
ITS: Spread distance between toes 2 and 4
MSCs: Mesenchymal stem cells
MSCs: Mesenchymal stem/stromal cell
*n*: Number of animals per group
NEPT: Normal extended postural thrust;
NGC: Nerve guidance conduit
OM: Olfactory mucosa
OM-MSCs: Olfactory mucosa mesenchymal stem/stromal cells
PBS: Phosphate-buffered saline solution
PL: Print length
PNI: Peripheral nerve injuries
R: Suture of the nerve ends to the Reaxon® NGC
ROM: Suture of the nerve ends to the Reaxon® NGC and administration of OM-MSCs suspended in Matrigel®
RQ: Relative quantification
RT-PCR: Reverse transcriptase polymerase chain reaction
SEM: Scanning electron microscopy
SFI: Sciatic functional index
SPM: Statistical parametric mapping
SSI: Static sciatic index
TS: Spread distance between toes 1 and 5
UC: Uninjured control
WRL: Withdrawal reflex latency.

## Supplementary Materials

Supplementary Materials Table S1: list of genes under study with the respective amplicon context sequence, ensemble gene ID, and PCR product length. Table S2: corrected absorbance in the different groups assessed by the PrestoBlue® viability assay, in different time points. Results presented as mean ± SEM. Table S3: statistical differences in the corrected absorbance identified between different groups: (a) 24 h; (b) 72 h; (c) 120 h; (d) 168 h; (e) 216 h (ns = no statistically significant differences). Table S4: values of functional deficit (%) obtained performing the EPT test. These tests were performed preoperatively (T0) and 1 and 2 weeks after neurotmesis (T1 and T2) and from there every two weeks until week 20 (T20). Results are presented as mean and SD (*n* = number of animals per group). Table S5: statistical differences observed in the EPT test at 20 weeks (ns = no statistically significant differences). Table S6: WRL values in seconds (s) obtained performing the WRL test. These tests were performed preoperatively (T0) and 1 and 2 weeks after neurotmesis (T1 and T2) and from there every two weeks until week 20 (T20). Results are presented as mean and SD (*n* = number of animals per group). Table S7: statistical differences observed in the EPT test at 20 weeks (ns = no statistically significant differences). Table S8: results of functional recovery through the SFI. These tests were performed preoperatively (T0) and 1 and 2 weeks after neurotmesis (T1 and T2) and from there every two weeks until week 20 (T20). Results are presented as mean and SD (*n* = number of animals per group. Table S9: statistical differences observed in the SFI test at 20 weeks (ns = no statistically significant differences). Table S10: results of functional recovery through the SSI. These tests were performed preoperatively (T0) and 1 and 2 weeks after neurotmesis (T1 and T2) and from there every two weeks until week 20 (T20). Results are presented as mean and SD (*n* = number of animals per group). Table S11: statistical differences observed in the SSI test at 20 weeks (ns = no statistically significant differences). Table S12: stereological quantitative assessment. The different parameters considered were evaluated in the regenerated sciatic nerve at week 20 (T20) after neurotmesis. Results are presented as mean and SD (*n* = number of animals per group). Table S13: statistical differences in the nerve stereological analysis: (a) density of fibers; (b) total number of fibers; (c) axon diameter; (d) fiber diameter; (e) myelin thickness; (f) g-ratio; (g) cross-sectional area (ns = no statistically significant differences). Table S14: statistically significant differences in the evaluation of the tibial cranial muscles: (a) individual fiber area; (b) minimum Feret's diameter of the muscle fibers (ns = no statistically significant differences).
