# Supplementary material for: Combined Use of Chitosan and Olfactory Mucosa Mesenchymal Stem/Stromal Cells to Promote Peripheral Nerve Regeneration In Vivo
Source: Stem Cells Int. 2021 Jan 2;2021:6613029. doi: 10.1155/2021/6613029 (PMC7801080; doi:10.1155/2021/6613029)
Supplement: Supplementary Materials — Table S1: list of genes under study with the respective amplicon context sequence, ensemble gene ID, and PCR product length. Table S2: corrected absorbance in the different groups assessed by the PrestoBlue® viability assay, in different time points. Results presented as mean ± SEM. Table S3: statistical differences in the corrected absorbance identified between different groups: (a) 24 h; (b) 72 h; (c) 120 h; (d) 168 h; (e) 216 h (ns = no statistically significant differences). Table S4: values of functional deficit (%) obtained performing the EPT test. These tests were performed preoperatively (T0) and 1 and 2 weeks after neurotmesis (T1 and T2) and from there every two weeks until week 20 (T20). Results are presented as mean and SD (n = number of animals per group). Table S5: statistical differences observed in the EPT test at 20 weeks (ns = no statistically significant differences). Table S6: WRL values in seconds (s) obtained performing the WRL test. These tests were performed preoperatively (T0) and 1 and 2 weeks after neurotmesis (T1 and T2) and from there every two weeks until week 20 (T20). Results are presented as mean and SD (n = number of animals per group). Table S7: statistical differences observed in the EPT test at 20 weeks (ns = no statistically significant differences). Table S8: results of functional recovery through the SFI. These tests were performed preoperatively (T0) and 1 and 2 weeks after neurotmesis (T1 and T2) and from there every two weeks until week 20 (T20). Results are presented as mean and SD (n = number of animals per group. Table S9: statistical differences observed in the SFI test at 20 weeks (ns = no statistically significant differences). Table S10: results of functional recovery through the SSI. These tests were performed preoperatively (T0) and 1 and 2 weeks after neurotmesis (T1 and T2) and from there every two weeks until week 20 (T20). Results are presented as mean and SD (n = number of animals per group). Table S11: statis [file 6613029.f1.docx]

**Table S1** - List of genes under study with respective amplicon context sequence, ensemble gene ID and PCR product length.

| **Marker Group** | **Marker** | **Gene Symbol** | **Amplicon Context Sequence** | **Ensembl Gene ID** | **PCR product** |  |
| --- | --- | --- | --- | --- | --- | --- |
| **Housekeeping** | **β-actin** | **Actb** | CGTCCACCCGCGAGTACAACCTTCTTGCAGCTCCTCCGTCGCCGGTCCACACCCGCCACCAGTTCGCCATGGATGACGATATCGCTGCGCTCGTCGTCGACAAC | ENSRNOG00000034254 | 74 bp |  |
|  |  |  |  |  |  |  |
| **Glial cells** | **Aldh1l1** | **Aldh1l1** | TTGGATGCGAATCCTGCCGAACGTCCCAGAGGTGGAAGACTCTACAGATTTCTTCAAGTCAGGAGCTGCTTCTGTAGATGTTGTGAGGCTGGTGAGGAAGTGAAGGAGCTGTGTGACGG | ENSRNOG00000047023 | 90 bp |  |
|  | **CD40** | **CD40** | TGCTCTGATCTCGCTCTGCAATGCTGCCTTTGCCTCAGCTGTGCGCGCTCTGGGGCTGCTTGTTGACAGCGGTCCATCTAGGACAGTGTGTTACGTGCAGTGACAAACAGTACCTCCAAGGTGGCGAGTGCTGCGATTTGTGCCAGCCGG | ENSRNOG00000018488 | 120 bp |  |
|  | **N-cadherin** | **Cdh2** | CATTGATATCAAGTAAATAGATTTGCAGCGTTCCTGTTCCACTCATAGGAGGGATTCCATTGTCAGAAGCAAGGAAGGTAGCATTATAGATGTTGTTTTGACATTTGGCGACTCTCTGTCCAGAACAGCGATAGTGGTGATCT | ENSRNOG00000015602 | 114 bp |  |
|  | **NG2** | **Cspg4** | CTTTTCTACCTCCGCAAACGCAACAAGACAGGTAAGCATGATGTCCAGGTGTTGACCGCCAAACCCCGCAATGGCCTAGCTGGTGACACAGAGACCTTTC | ENSRNOG00000017208 | 70 bp |  |
|  | **Neuromodulin** | **GAP-43** | GGAAAGGAGAGAAGGCAGGAAGAAGGCAGGGGAAGATACCACCATGCTGTGCTGTATGAGAAGAACCAAACAGGTTGAAAAGAATGATGAGGACCAAAAGATTGAACAAGATGGTGTCAAACCGGAGGATAAGGCTCATAAGGC | ENSRNOG00000001528 | 114 bp |  |
|  | **GFAP** | **GFAP** | ACTTTCAAACAGGATGGACGCTTAAAGTCTAGGCGATACTCCGTACATGCATAAGAAAGGGAATGGGGCCCTTGGCTTGGAGAACAACAGCTAGTGGTTGTGGGGATGGAGGGCCTCAGAAGGATGGTTGTGGACTCT | ENSRNOG00000002919 | 108 bp |  |
|  | **Myelin protein P0** | **MPZ** | GGAGTCTCGCAAGGATAAGAAATAGCGGTTAGCGGGCCGGGCGGGGGGTCGGGGGTCTGCGATGGAGTCTTCCAAAGGCTCTCAGGTGGTGGTCATCGAGATGGAGCTTCGCAAAGATGAGCAGAGCTCGGAGCTCCGG | ENSRNOG00000003171 | 109 bp |  |
|  | **NCAM** | **NCAM** | CCACCATGTGCCCATCTAGAGTCTCTTGCTTCTCTGGTCGAGTCCACGATGCCTTTTCTTCACTGCTGATGTTTCGGGTGGACGTTCTCCAGGTGATGGAAGGAATGGGGTCTCCGGAGGCTTCACATGTCAGAGTGACTTGCTCCTCTAGTTCCATGGCTGTCTGATTCTCTACATAGGTGA | ENSRNOG00000031890 | 153 bp |  |
|  | **Nestin** | **Nes** | GATGAGGATAGAGCCCAAGCAGGTGAACAAGACTCCATAGAGGTGACCCTTGGGTTAGAGGCTGCCAGAACTGGACTGGAACTCGAGCAGGAAGTGGTAGGGCTAGAGGAC | ENSRNOG00000018681 | 81 bp |  |
|  | **Occludin** | **Ocln** | GAAGTCATCCACGGACAAGGTCAGAGGAATCTCCTGGGCTACTTCAGGTACCAGAGGCGGTGACTTATACAAAGAGTCTGGGTAGGATCTCTTGCCATTCACTTTGCCGTTGGAGGAGTAGGCCATCGGACT | ENSRNOG00000018297 | 102 bp |  |
|  | **Olig3** | **Olig3** | GCGTCATCTCCGGACATGGATGAGATGTACCTAAGAGACCACCACCACCGCCACCACCACCACCATCAGGAGAGCCGTCTTAACTCGGTCTCATCCA | ENSRNOG00000012057 | 67 bp |  |
|  | **Sox10** | **Sox10** | GCAGGTATTGGTCCAGCTCAGTCACATCAAAGGTCTCCATGTTGGACATTACCTCATGGCTGATCTCCCCGATGTCCACATTGCCGAAGTCGATGTGGGGCTTCCCGCCCTCCCCCAAG | ENSRNOG00000011305 | 89 bp |  |
|  | **Sox2** | **Sox2** | GCAGTACAACTCCATGACCAGCTCGCAGACCTACATGAACGGCTCGCCCACCTACAGCATGTCCTACTCGCAGCAGGGCACCCCCGGTATGGCGCTGGGCTCCATGGGCTCTGTGGTCAAGTCCGAGGCCAGTTCC | ENSRNOG00000012199 | 106 bp |  |
| **Neurons** | **MASH1** | **Ascl1** | AGTTAAAAGGGAAGAAACGCGCAGAAGGAGGCAGAGTTGTCTAGCAGCAACGGAGGAAGGTGCCTGGGTGATCGTCTTCCCTCTGCGCCGCCTCCTCCCTCCCCT | ENSRNOG00000004294 | 75 bp |  |
|  | **Doublecortin** | **Dcx** | TACATTGACCGACCAGTTGGGATTGACATTCTTGGTATACTCAACCTTTTTAAAGAAGTTGTCTGAGGAACAGACATAGCTTTCCCCTTCTTCCAGTTCATCCATGCTTCCAATCTTTCT | ENSRNOG00000047712 | 90 bp |  |
|  | **MAP2** | **MAP2** | TTCCTCCTCCTCTCTGTATGGAAATCCATTGGCGCTGCGGCTCAGCCCTTCCCCTGAGCCACCCTGGTCCTTCATCTCTGGCGAGTGGGGGTGTGCAGC | ENSRNOG00000011841 | 69 bp |  |
|  | **NeuroD1** | **NueroD1** | CCCGGGAATGGTGAAACTGACGTGCCTCTAATCGTGAAAGATGGCATTAAGCTGGGCACTCATGACTCGCTCATGATGCGAATGGCTATCGAAAGACATAATATTGTCTATGGGGATCTCACAGCGAGGAGCAGCGGCACCCGAGGAG | ENSRNOG00000005609 | 118 bp |  |
|  | **NeuN** | **Rbfox3** | GGTCGGGGTCCCTGAACCGGAAGGGGATGTTGGAGACATGTAGTCGTTTGGGCTGCTGCTTCTCCGTGGGGTCGGAAGGGTGGAGTTGCTGGCTATCTGTCTGTGCTGCTTCAT | ENSRNOG00000003386 | 84 bp |  |
|  | **Synaptophysin** | **Syn** | GGGGCTGCCCAGCCTGTCTCCTTGAACACGAACCATAAGTTGCCAACCCAGAGCACCAGGTTCAGGAAGCCAAACACCACTGAGGTGTTGAGTCCTGAAGTCACAGGGTCCCTCAGTTCCTTGCATGTGTTCCCTGTCTGGCGG | ENSRNOG00000010223 | 114 bp |  |
|  | **Cdk5r1** | **Cdk5r1** | ACCTGTCTGTACCTCTCCTATTCCTACATGGGCAATGAAATCTCCTACCCGCTCAAGCCCTTCCTGGTGGAGAGCTGCAAGGAAGCCTTTTGGGACCGTTGCCTCTCAGTTATCAACCTCATGAGCTCCAAGATGCTGCAGATCAATGCT | ENSRNOG00000021685 | 120 bp |  |
|  | **Cript** | **Cript** | AAACTACAAGCAGACTTCCGTCTAGGCTGTTGAAGATTCCAACTCTACGGTTGTATTTGTGCTTCAGCTTTTCAAGCGTAACACGGATGTCCATCTCACAAAATGACCTGTTTGAGGATGAAT | ENSRNOG00000015215 | 93 bp |  |
|  | **Tubulin beta-3** | **Tubb3** | GGCCAAGACAAGCAGCATCTGTCCCCCAGAGCCATCTAGCTACTGACACTGCCCCCAGCTTACCTTCCTACCAGCTCTTCAGGGCACCCTAGGGCTCCCGGGTTAAA | ENSRNOG00000017209 | 77 bp |  |

**Table S2** – Corrected absorbance in the different groups assessed by PrestoBlue® viability assay, in different timepoints. Results presented as Mean ± SEM

|  | **OM-MSCs + Basal Medium + Reaxon®** | **OM-MSCs + Basal Medium + Matrigel®** | **OM-MSCs + Basal Medium** | **OM-MSCs + Basal Medium + DMSO 10%** |
| --- | --- | --- | --- | --- |
| **24h** | **0.017 + 0.001** | **0.027 + 0.001** | **0.043 + 0.001** | **0.010 + 0.001** |
| **72h** | **0.124 + 0.010** | **0.153 + 0.015** | **0.114 + 0.003** | **0.009 + 0.001** |
| **120h** | **0.284 + 0.004** | **0.292 + 0.004** | **0.270 + 0.005** | **0.005 + 0.003** |
| **168h** | **0.290 + 0.003** | **0.294 + 0.002** | **0.283 + 0.005** | **0.003 + 0.003** |
| **216h** | **0.300 + 0.002** | **0.303 + 0.002** | **0.297 + 0.003** | **0.001 + 0.002** |

| **24h** | | | |  |
| --- | --- | --- | --- | --- |
|  | **OM-MSCs + Basal Medium + Reaxon®** | **OM-MSCs + Basal Medium + Matrigel®** | **OM-MSCs + Basal Medium** | **OM-MSCs + Basal Medium + DMSO** |
| **OM-MSCs + Basal Medium + Reaxon®**  **a)** |  | ******** | ******** | ******* |
| **OM-MSCs + Basal Medium + Matrigel®** |  |  | ******** | ******** |
| **OM-MSCs + Basal Medium** |  |  |  | ******** |
| **OM-MSCs + Basal Medium + DMSO** |  |  |  |  |

**Table S3**: Statistical differences in the corrected absorbance identified between different groups: **a)** 24h; **b)** 72h; **c)** 120h; **d)** 168h; **e)** 216h. (*ns* = no statistically significant differences).

| **72h** | | | |  |
| --- | --- | --- | --- | --- |
| **b)** | **OM-MSCs + Basal Medium + Reaxon®** | **OM-MSCs + Basal Medium + Matrigel®** | **OM-MSCs + Basal Medium** | **OM-MSCs + Basal Medium + DMSO** |
| **OM-MSCs + Basal Medium + Reaxon®** |  | **ns** | **ns** | ******** |
| **OM-MSCs + Basal Medium + Matrigel®** |  |  | ***** | ******** |
| **OM-MSCs + Basal Medium** |  |  |  | ******** |
| **OM-MSCs + Basal Medium + DMSO** |  |  |  |  |

| **120h** | | | |  |
| --- | --- | --- | --- | --- |
| **c)** | **OM-MSCs + Basal Medium + Reaxon®** | **OM-MSCs + Basal Medium + Matrigel®** | **OM-MSCs + Basal Medium** | **OM-MSCs + Basal Medium + DMSO** |
| **OM-MSCs + Basal Medium + Reaxon®** |  | **ns** | **ns** | ******** |
| **OM-MSCs + Basal Medium + Matrigel®** |  |  | ****** | ******** |
| **OM-MSCs + Basal Medium** |  |  |  | ******** |
| **OM-MSCs + Basal Medium + DMSO** |  |  |  |  |

| **168h** | | | |  |
| --- | --- | --- | --- | --- |
|  | **OM-MSCs + Basal Medium + Reaxon®** | **OM-MSCs + Basal Medium + Matrigel®** | **OM-MSCs + Basal Medium** | **OM-MSCs + Basal Medium + DMSO** |
| **OM-MSCs + Basal Medium + Reaxon®** |  | **ns** | **ns** | ******** |
| **OM-MSCs + Basal Medium + Matrigel®** |  |  | **ns** | ******** |
| **OM-MSCs + Basal Medium** |  |  |  | ******** |
| **OM-MSCs + Basal Medium + DMSO** |  |  |  |  |

| **216h** | | | |  |
| --- | --- | --- | --- | --- |
|  | **OM-MSCs + Basal Medium + Reaxon®** | **OM-MSCs + Basal Medium + Matrigel®** | **OM-MSCs + Basal Medium** | **OM-MSCs + Basal Medium + DMSO** |
| **OM-MSCs + Basal Medium + Reaxon®** |  | **ns** | **ns** | ******** |
| **OM-MSCs + Basal Medium + Matrigel®** |  |  | **ns** | ******** |
| **OM-MSCs + Basal Medium** |  |  |  | ******** |
| **OM-MSCs + Basal Medium + DMSO** |  |  |  |  |

**e)**

**d)**

**Table S4**: Values of functional deficit (%) obtained performing the EPT test. These tests were performed preoperatively (T0), 1 and 2 weeks after neurotmesis (T1 and T2) and from there every two weeks until week 20 (T20). Results are presented as mean and SD. (*n* = number of animals per group).

| **EPT** | |  | **Time** | | | | | | | | | | |
| --- | --- | --- | --- | --- | --- | --- | --- | --- | --- | --- | --- | --- | --- |
|  |  | **T0** | **T1** | **T2** | **T4** | **T6** | **T8** | **T10** | **T12** | **T14** | **T16** | **T18** | **T20** |
| **Group 1: UC  (*n*= 30)** | Mean | 7.29 | 7.29 | 7.29 | 7.29 | 7.29 | 7.29 | 7.29 | 7.29 | 7.29 | 7.29 | 7.29 | 7.29 |
|  | SD | 5.12 | 5.12 | 5.12 | 5.12 | 5.12 | 5.12 | 5.12 | 5.12 | 5.12 | 5.12 | 5.12 | 5.12 |
| **Group 2: EtE (*n* = 5)** | Mean | 7.20 | 90.00 | 90.00 | 85.60 | 79.20 | 76.60 | 69.20 | 59.00 | 52.40 | 44.00 | 40.60 | 39.20 |
|  | SD | 0,45 | 2.12 | 2.12 | 4.45 | 7.53 | 6.07 | 8.44 | 6.28 | 7.92 | 5.48 | 5.73 | 3.35 |
| **Group 3: R (*n* = 6)** | Mean | 6.69 | 100.00 | 100.00 | 83.70 | 83.32 | 76.46 | 74.13 | 46.05 | 40.44 | 36.87 | 23.91 | 24.43 |
|  | SD | 2.51 | 0.00 | 0.00 | 6.63 | 4.47 | 3.82 | 4.57 | 11.06 | 8.08 | 7.43 | 11.27 | 8.11 |
| **Group 4: EtER (*n* = 6)** | Mean | 4.90 | 100.00 | 92.52 | 90.85 | 80.40 | 76.40 | 70.99 | 33.15 | 28.34 | 25.46 | 25.13 | 22.59 |
|  | SD | 9.22 | 0.00 | 3.24 | 1.87 | 9.09 | 7.68 | 15.30 | 10.56 | 11.62 | 4.16 | 3.83 | 7.22 |
| **Group 5: ROM (*n* = 6)** | Mean | 7.99 | 100.00 | 89.53 | 86.41 | 83.45 | 69.13 | 67.70 | 35.35 | 31.04 | 18.21 | 17.92 | 17.49 |
|  | SD | 4.14 | 0,00 | 6.39 | 4.15 | 9.89 | 7.79 | 8.67 | 8.40 | 10.32 | 11.35 | 11.62 | 11.66 |
| **Group 6: EtEROM (*n* = 7)** | Mean | 9.54 | 100.00 | 100.00 | 91.79 | 85.34 | 77.27 | 69.79 | 54.15 | 41.16 | 31.46 | 28.05 | 20.90 |
|  | SD | 5.11 | 0.00 | 0.00 | 4.34 | 3.36 | 10.14 | 8.19 | 12.06 | 14.41 | 15.91 | 12.52 | 7.80 |

**Table S5**: Statical differences observed in EPT test at 20 weeks. (*ns* = no statistically significant differences).

| **EPT** | | | | | | |
| --- | --- | --- | --- | --- | --- | --- |
|  | **UC** | **EtE** | **R** | **EtER** | **ROM** | **EtEROM** |
| **UC** |  | ******** | ******** | ******** | ****** | ******* |
| **EtE** |  |  | ****** | ****** | ******** | ******* |
| **R** |  |  |  | **ns** | **ns** | **ns** |
| **EtER** |  |  |  |  | **ns** | **ns** |
| **ROM** |  |  |  |  |  | **ns** |
| **EtEROM** |  |  |  |  |  |  |

**Table S6:** WRL values in seconds (s) obtained performing WRL test. These tests were performed preoperatively (T0), 1 and 2 weeks after neurotmesis (T1 and T2) and from there every two weeks until week 20 (T20). Results are presented as mean and SD. (*n* = number of animals per group).

| **WRL** | |  | **Time** | | | | | | | | | | |
| --- | --- | --- | --- | --- | --- | --- | --- | --- | --- | --- | --- | --- | --- |
|  |  | **T0** | **T1** | **T2** | **T4** | **T6** | **T8** | **T10** | **T12** | **T14** | **T16** | **T18** | **T20** |
| **Group 1: UC  (*n*= 30)** | Mean | 2.39 | 2.39 | 2.39 | 2.39 | 2.39 | 2.39 | 2.39 | 2.39 | 2.39 | 2.39 | 2.39 | 2.39 |
|  | SD | 1.17 | 1.17 | 1.17 | 1.17 | 1.17 | 1.17 | 1.17 | 1.17 | 1.17 | 1.17 | 1.17 | 1.17 |
| **Group 2: EtE (*n* = 5)** | Mean | 4.28 | 12,00 | 12,00 | 11.85 | 10.48 | 10.45 | 10.22 | 9.82 | 9.26 | 8.78 | 8.57 | 7.5 |
|  | SD | 0.86 | 0,00 | 0,00 | 0.34 | 2.86 | 2.12 | 2.5 | 2.02 | 3.07 | 2.22 | 0.98 | 0.87 |
| **Group 3: R (*n* = 6)** | Mean | 1.94 | 12,00 | 11.89 | 8.72 | 7.44 | 7.28 | 6.94 | 5.89 | 4.72 | 3.83 | 3.33 | 3.22 |
|  | SD | 0.86 | 0,00 | 0.27 | 1.93 | 2.86 | 3.57 | 1.73 | 3.54 | 2.26 | 1.74 | 1.43 | 0.93 |
| **Group 4: EtER (*n* = 6)** | Mean | 2.12 | 12,00 | 11.56 | 10.78 | 8.61 | 6.5 | 5.78 | 4.44 | 4.17 | 3.83 | 3.06 | 2.5 |
|  | SD | 0.86 | 0,00 | 1.09 | 1.71 | 2.04 | 2.07 | 1.6 | 2.39 | 1.17 | 1.01 | 0.83 | 0.81 |
| **Group 5: ROM (*n* = 6)** | Mean | 2.05 | 12,00 | 12,00 | 11.19 | 9.95 | 7.95 | 5.95 | 5.86 | 4.19 | 3.24 | 3.19 | 3.05 |
|  | SD | 0.83 | 0,00 | 2.63 | 0.86 | 2.02 | 2.98 | 2.35 | 1.75 | 0.94 | 0.85 | 0.94 | 1.19 |
| **Group 6: EtEROM (*n* = 7)** | Mean | 1.93 | 12,00 | 11.28 | 10,00 | 9.5 | 9.39 | 6.89 | 5.06 | 4.28 | 3.94 | 3.83 | 3.67 |
|  | SD | 0.83 | 0,00 | 1.16 | 2.43 | 2.67 | 1.6 | 3.28 | 1.73 | 1.48 | 1.16 | 1.77 | 2.01 |

**Table S7**: Statical differences observed in EPT test at 20 weeks. (*ns* = no statistically significant differences).

| **WRL** | | | | | | |
| --- | --- | --- | --- | --- | --- | --- |
|  | **UC** | **EtE** | **R** | **EtER** | **ROM** | **EtEROM** |
| **UC** |  | ******** | **ns** | **ns** | **ns** | **ns** |
| **EtE** |  |  | ******** | ******** | ******** | ******** |
| **R** |  |  |  | **ns** | **ns** | **ns** |
| **EtER** |  |  |  |  | **ns** | **ns** |
| **ROM** |  |  |  |  |  | **ns** |
| **EtEROM** |  |  |  |  |  |  |

**Table S8:** Results of functional recovery through the SFI. These tests were performed preoperatively (T0), 1 and 2 weeks after neurotmesis (T1 and T2) and from there every two weeks until week 20 (T20). Results are presented as mean and SD. (*n* = number of animals per group

| **SFI** | |  | **Time** | | | | | | | | | | |
| --- | --- | --- | --- | --- | --- | --- | --- | --- | --- | --- | --- | --- | --- |
|  |  | **T0** | **T1** | **T2** | **T4** | **T6** | **T8** | **T10** | **T12** | **T14** | **T16** | **T18** | **T20** |
| **Group 1: UC  (*n*= 30)** | Mean | -1.45 | -1.45 | -1.45 | -1.45 | -1.45 | -1.45 | -1.45 | -1.45 | -1.45 | -1.45 | -1.45 | -1.45 |
|  | SD | 11.12 | 11.12 | 11.12 | 11.12 | 11.12 | 11.12 | 11.12 | 11.12 | 11.12 | 11.12 | 11.12 | 11.12 |
| **Group 2: EtE (*n* = 5)** | Mean | 0.06 | -86.73 | -86.73 | -81.15 | -71.8 | -67.1 | -61.98 | -59.59 | -58.39 | -54.19 | -53.99 | -46.56 |
|  | SD | 3.91 | 4.22 | 4.22 | 17.11 | 8.12 | 6.79 | 11.39 | 25.98 | 3.2 | 20.18 | 30.3 | 14.54 |
| **Group 3: R (*n* = 6)** | Mean | -6.41 | -73.61 | -63.93 | -63.58 | -60.33 | -60.04 | -53.63 | -51.22 | -38.9 | -37.12 | -30.82 | -29.46 |
|  | SD | 4.68 | 8.76 | 18.23 | 23.51 | 9.3 | 12.61 | 20.45 | 11.71 | 23.66 | 22.19 | 20.1 | 21.75 |
| **Group 4: EtER (*n* = 6)** | Mean | 4.51 | -59.14 | -53.18 | -52.75 | -47.97 | -44.31 | -38.59 | -35.53 | -30.67 | -27.78 | -23.85 | -20.01 |
|  | SD | 15.16 | 17.01 | 12,00 | 14.3 | 12.46 | 25.69 | 14.22 | 24.62 | 13.96 | 10.8 | 14.05 | 17.12 |
| **Group 5: ROM (*n* = 6)** | Mean | -7.46 | -65.38 | -56.66 | -56.59 | -55.29 | -53.24 | -52.66 | -46.48 | -25.59 | -22.45 | -15.3 | -13.41 |
|  | SD | 5.09 | 9.09 | 13.33 | 14.46 | 16.21 | 20.44 | 21.74 | 20.51 | 13.94 | 9.59 | 11.27 | 12.57 |
| **Group 6: EtEROM (*n* = 7)** | Mean | 3.32 | -71.16 | -51.53 | -55.17 | -55.07 | -52.44 | -50.59 | -49.83 | -42.43 | -27.03 | -20.88 | -13.89 |
|  | SD | 16.44 | 13.48 | 18.3 | 17.77 | 12.56 | 24.98 | 11.97 | 16.1 | 19,00 | 10.61 | 9.46 | 9.94 |

**Table S9**: Statical differences observed in SFI test at 20 weeks. (*ns* = no statistically significant differences).

| **SFI** | | | | | | |
| --- | --- | --- | --- | --- | --- | --- |
|  | **UC** | **EtE** | **R** | **EtER** | **ROM** | **EtEROM** |
| **UC** |  | ******** | ******* | ***** | **ns** | **ns** |
| **EtE** |  |  | **ns** | ***** | ****** | ****** |
| **R** |  |  |  | **ns** | **ns** | **ns** |
| **EtER** |  |  |  |  | **ns** | **ns** |
| **ROM** |  |  |  |  |  | **ns** |
| **EtEROM** |  |  |  |  |  |  |

**Table S10:** Results of functional recovery through the SSI. These tests were performed preoperatively (T0), 1 and 2 weeks after neurotmesis (T1 and T2) and from there every two weeks until week 20 (T20). Results are presented as mean and SD. (*n* = number of animals per group).

| **SSI** | |  | **Time** | | | | | | | | | | |
| --- | --- | --- | --- | --- | --- | --- | --- | --- | --- | --- | --- | --- | --- |
|  |  | **T0** | **T1** | **T2** | **T4** | **T6** | **T8** | **T10** | **T12** | **T14** | **T16** | **T18** | **T20** |
| **Group 1: UC  (*n*= 30)** | Mean | 1.98 | 1.98 | 1.98 | 1.98 | 1.98 | 1.98 | 1.98 | 1.98 | 1.98 | 1.98 | 1.98 | 1.98 |
|  | SD | 11.87 | 11.87 | 11.87 | 11.87 | 11.87 | 11.87 | 11.87 | 11.87 | 11.87 | 11.87 | 11.87 | 11.87 |
| **Group 2: EtE (*n* = 5)** | Mean | -5.78 | -74.44 | -73.92 | -70.22 | -68.04 | -59.97 | -52.47 | -46.56 | -44.21 | -43.04 | -41.91 | -33.72 |
|  | SD | 12.91 | 6.83 | 6.82 | 6.44 | 11.02 | 2.64 | 27.43 | 16.79 | 15.34 | 12.31 | 24.55 | 15.72 |
| **Group 3: R (*n* = 6)** | Mean | -1.16 | -74.83 | -68.41 | -68.1 | -58.16 | -58.05 | -54.14 | -46.71 | -33.34 | -26.29 | -21.07 | -19.93 |
|  | SD | 5.83 | 9.48 | 11.06 | 18.15 | 11.07 | 15.57 | 25.15 | 14.13 | 27,00 | 20.68 | 21.03 | 17.32 |
| **Group 4: EtER (*n* = 6)** | Mean | 9.06 | -56.88 | -52.47 | -52.3 | -52.13 | -43.12 | -37.35 | -34.22 | -26.88 | -25.43 | -19.26 | -15.75 |
|  | SD | 14.19 | 21.86 | 16.13 | 11.87 | 12.95 | 21.78 | 18.93 | 26.39 | 18.09 | 17.49 | 14.39 | 15.03 |
| **Group 5: ROM (*n* = 6)** | Mean | -1.43 | -67.49 | -59.99 | -59.18 | -54.31 | -53,00 | -50.1 | -46.94 | -24.03 | -18.34 | -11.64 | -9.85 |
|  | SD | 5.37 | 12.07 | 14.97 | 14.46 | 18.56 | 23.73 | 22.13 | 23.17 | 15.5 | 11.1 | 12.26 | 14.06 |
| **Group 6: EtEROM (*n* = 7)** | Mean | 8.5 | -69.1 | -62.61 | -56.11 | -50.97 | -48.39 | -45.93 | -45.07 | -40.72 | -24.18 | -17.05 | -10.39 |
|  | SD | 14.66 | 14.57 | 5.63 | 15.8 | 23.57 | 20.29 | 14.06 | 17.5 | 17.21 | 11.02 | 8.45 | 8.99 |

**Table S11:** Statical differences observed in SSI test at 20 weeks. (*ns* = no statistically significant differences).

| **SSI** | | | | | | |
| --- | --- | --- | --- | --- | --- | --- |
|  | **UC** | **EtE** | **R** | **EtER** | **ROM** | **EtEROM** |
| **UC** |  | ******** | ****** | ***** | **ns** | **ns** |
| **EtE** |  |  | **ns** | **ns** | ***** | **ns** |
| **R** |  |  |  | **ns** | **ns** | **ns** |
| **EtER** |  |  |  |  | **ns** | **ns** |
| **ROM** |  |  |  |  |  | **ns** |
| **EtEROM** |  |  |  |  |  |  |

**Table S12**: Stereological quantitative assessment. The different parameters considered were evaluated in the regenerated sciatic nerve at week 20 (T20) after neurotmesis. Results are presented as mean and SD (*n* = number of animals per group).

| **Stereological Quantitative Assessment** | | **Density** | **Total number** | **Axon diameter (d)** | **Fiber diameter (D)** | **Myelin thickness (M)** | **M/d** | **D/d** | **d/D (g-ratio)** | **Cross-sectional area (mm^2^)** |  |
| --- | --- | --- | --- | --- | --- | --- | --- | --- | --- | --- | --- |
|  |  |  |  |  |  |  |  |  |  |  |  |
| **Group 1: UC  (*n*= 4)** | Mean | 10167 | 10200 | 5.00 | 8.28 | 1.64 | 0.35 | 1.71 | 0.60 | 1.005 |  |
|  | SD | 490 | 1355 | 0.39 | 0.40 | 0.05 | 0.03 | 0.07 | 0.02 | 0.1258 |  |
| **Group 2: EtE (*n* = 5)** | Mean | 30072 | 17423 | 2.37 | 3.77 | 0.70 | 0.35 | 1.70 | 0.60 | 0.6100 |  |
|  | SD | 5443 | 2217 | 0.13 | 0.21 | 0.05 | 0.02 | 0.03 | 0.01 | 0.2115 |  |
| **Group 3: R (*n* = 6)** | Mean | 27105 | 15724 | 2.22 | 3.63 | 0.70 | 0.39 | 1.78 | 0.59 | 0.5875 |  |
|  | SD | 4318 | 5145 | 0.15 | 0.21 | 0.04 | 0.02 | 0.03 | 0.01 | 0.2021 |  |
| **Group 4: EtER (*n* = 6)** | Mean | 24886 | 14115 | 2.53 | 4.08 | 0.78 | 0.38 | 1.75 | 0.60 | 0.5667 |  |
|  | SD | 2634 | 2436 | 0.18 | 0.33 | 0.08 | 0.04 | 0.07 | 0.02 | 0.0709 |  |
| **Group 5: ROM (*n* = 6)** | Mean | 27196 | 16785 | 2.30 | 3.68 | 0.69 | 0.36 | 1.73 | 0.60 | 0.6240 |  |
|  | SD | 3258 | 3371 | 0.09 | 0.07 | 0.01 | 0.03 | 0.06 | 0.01 | 0.1450 |  |
| **Group 6: EtEROM (*n* = 7)** | Mean | 24277 | 16134 | 2.31 | 3.73 | 0.71 | 0.37 | 1.73 | 0.60 | 0.6914 |  |
|  | SD | 4862 | 3891 | 0.15 | 0.23 | 0.05 | 0.03 | 0.06 | 0.02 | 0.2441 |  |

| **Density** | | | | | | |
| --- | --- | --- | --- | --- | --- | --- |
|  | **UC** | **EtE** | **R** | **EtER** | **ROM** | **EtEROM** |
| **UC** |  | ******** | ******** | ******** | ******** | ******** |
| **EtE** |  |  | ****** | ******** | ******* | ******** |
| **R** |  |  |  | **ns** | **ns** | **ns** |
| **EtER** |  |  |  |  | **ns** | **ns** |
| **ROM** |  |  |  |  |  | **ns** |
| **EtEROM** |  |  |  |  |  |  |

**Table S13:** Statistical differences in the nerve stereological analysis: **a)** Density of fibers; **b)** Total number of fibers; **c)** Axon diameter; **d)** Fiber diameter; **e)** Myelin thickness; **f)** g-ratio; **g)** Cross sectional area. (*ns* = no statistically significant differences).

**a)**

| **Total number** | | | | | | |
| --- | --- | --- | --- | --- | --- | --- |
|  | **UC** | **EtE** | **R** | **EtER** | **ROM** | **EtEROM** |
| **UC** |  | ****** | **ns** | **ns** | **ns** | **ns** |
| **EtE**  **b)** |  |  | **ns** | ***** | **ns** | **ns** |
| **R** |  |  |  | **ns** | **ns** | **ns** |
| **EtER** |  |  |  |  | **ns** | **ns** |
| **ROM** |  |  |  |  |  | **ns** |
| **EtEROM** |  |  |  |  |  |  |

| **Axon diameter (d)** | | | | | | |
| --- | --- | --- | --- | --- | --- | --- |
|  | **UC** | **EtE** | **R** | **EtER** | **ROM** | **EtEROM** |
| **UC** |  | ******** | ******** | ******** | ******** | ******** |
| **EtE** |  |  | ***** | **ns** | **ns** | **ns** |
| **R**  **c)** |  |  |  | **ns** | **ns** | **ns** |
| **EtER** |  |  |  |  | **ns** | **ns** |
| **ROM** |  |  |  |  |  | **ns** |
| **EtEROM** |  |  |  |  |  |  |

| **Fiber diameter (D)** | | | | | | |
| --- | --- | --- | --- | --- | --- | --- |
|  | **UC** | **EtE** | **R** | **EtER** | **ROM** | **EtEROM** |
| **UC** |  | ******** | ******** | ******** | ******** | ******** |
| **EtE** |  |  | **ns** | ***** | **ns** | **ns** |
| **R**  **d)** |  |  |  | **ns** | **ns** | **ns** |
| **EtER** |  |  |  |  | **ns** | **ns** |
| **ROM** |  |  |  |  |  | **ns** |
| **EtEROM** |  |  |  |  |  |  |

| **Myelin thickness (M)** | | | | | | |
| --- | --- | --- | --- | --- | --- | --- |
|  | **UC** | **EtE** | **R** | **EtER** | **ROM** | **EtEROM** |
| **UC** |  | ******** | ******** | ******** | ******** | ******** |
| **EtE** |  |  | ******** | ******** | ******** | ******** |
| **R**  **e)** |  |  |  | **ns** | **ns** | **ns** |
| **EtER** |  |  |  |  | **ns** | **ns** |
| **ROM** |  |  |  |  |  | **ns** |
| **EtEROM** |  |  |  |  |  |  |

| **d/D (g-ratio)** | | | | | | |
| --- | --- | --- | --- | --- | --- | --- |
|  | **UC** | **EtE** | **R** | **EtER** | **ROM** | **EtEROM** |
| **UC** |  | ******** | **ns** | **ns** | **ns** | **ns** |
| **EtE** |  |  | ******** | ******** | ******** | ******** |
| **R**  **f)** |  |  |  | **ns** | **ns** | **ns** |
| **EtER** |  |  |  |  | **ns** | **ns** |
| **ROM** |  |  |  |  |  | **ns** |
| **EtEROM** |  |  |  |  |  |  |

| **Cross-sectional area (mm^2^)** | | | | | | |
| --- | --- | --- | --- | --- | --- | --- |
|  | **UC** | **EtE** | **R** | **EtER** | **ROM** | **EtEROM** |
| **UC** |  | ***** | ***** | ***** | ***** | **ns** |
| **EtE** |  |  | **ns** | **ns** | **ns** | **ns** |
| **R**  **g)** |  |  |  | **ns** | **ns** | **ns** |
| **EtER** |  |  |  |  | **ns** | **ns** |
| **ROM** |  |  |  |  |  | **ns** |
| **EtEROM** |  |  |  |  |  |  |

**Table S14:** Statically significant differences in the evaluation of the Tibial Cranial Muscles: **a**) individual fiber area; **b**) minimum Feret’s diameter of the muscle fibers (*ns* = no statistically significant differences).

| **Fibre Area** | | | | | | |
| --- | --- | --- | --- | --- | --- | --- |
|  | **UC** | **EtE** | **R** | **EtER** | **ROM** | **EtEROM** |
| **UC** |  | **ns** | ******** | ******** | ***** | ******* |
| **EtE**  **a)** |  |  | ******* | ******** | **ns** | **ns** |
| **R** |  |  |  | **ns** | ******* | **ns** |
| **EtER** |  |  |  |  | ******** | ***** |
| **ROM** |  |  |  |  |  | **ns** |
| **EtEROM** |  |  |  |  |  |  |

| **Minimum Feret’s Diameter** | | | | | | |
| --- | --- | --- | --- | --- | --- | --- |
|  | **UC** | **EtE** | **R** | **EtER** | **ROM** | **EtEROM** |
| **UC** |  | ******** | ******** | ******** | ******** | ******** |
| **EtE** |  |  | ******** | ******** | **ns** | ***** |
| **R**  **b)** |  |  |  | ***** | ******** | ******** |
| **EtER** |  |  |  |  | ******** | ****** |
| **ROM** |  |  |  |  |  | **ns** |
| **EtEROM** |  |  |  |  |  |  |
